# Immune response kinetics to SARS-CoV-2 infection and COVID-19 vaccination among nursing home residents—Georgia, October 2020–July 2022

**DOI:** 10.1371/journal.pone.0301367

**Published:** 2024-04-16

**Authors:** Zeshan A. Chisty, Deana D. Li, Melia Haile, Hollis Houston, Juliana DaSilva, Rahsaan Overton, Amy J. Schuh, Jenn Haynie, Jacob Clemente, Alicia G. Branch, Melissa M. Arons, Clarisse A. Tsang, Gerald J. Pellegrini, Julia Bugrysheva, Justina Ilutsik, Romy Mohelsky, Patricia Comer, Solomon B. Hundia, Hyungseok Oh, Matthew J. Stuckey, Caitlin D. Bohannon, Mohammed Ata Ur Rasheed, Monica Epperson, Natalie J. Thornburg, L. Clifford McDonald, Allison C. Brown, Preeta K. Kutty

**Affiliations:** 1 COVID-19 Response, Centers for Disease Control and Prevention, Atlanta, Georgia, United States of America; 2 Goldbelt C6, LLC, Chesapeake, Virginia, United States of America; 3 A.G. Rhodes Wesley Woods Heath and Rehab, Atlanta, Georgia, United States of America; 4 Emory Healthcare, Atlanta, Georgia, United States of America; 5 Emory University School of Medicine, Atlanta, Georgia, United States of America; 6 Coronavirus and Other Respiratory Viruses Division, Centers for Disease Control and Prevention, Atlanta, Georgia, United States of America; Qatar University, QATAR

## Abstract

**Background:**

Understanding the immune response kinetics to SARS-CoV-2 infection and COVID-19 vaccination is important in nursing home (NH) residents, a high-risk population.

**Methods:**

An observational longitudinal evaluation of 37 consenting vaccinated NH residents with/without SARS-CoV-2 infection from October 2020 to July 2022 was conducted to characterize the immune response to spike protein due to infection and/or mRNA COVID-19 vaccine. Antibodies (IgG) to SARS-CoV-2 full-length spike, nucleocapsid, and receptor binding domain protein antigens were measured, and surrogate virus neutralization capacity was assessed using Meso Scale Discovery immunoassays. The participant’s spike exposure status varied depending on the acquisition of infection or receipt of a vaccine dose. Longitudinal linear mixed effects modeling was used to describe trajectories based on the participant’s last infection or vaccination; the primary series mRNA COVID-19 vaccine was considered two spike exposures. Mean antibody titer values from participants who developed an infection post receipt of mRNA COVID-19 vaccine were compared with those who did not. In a subset of participants (n = 15), memory B cell (MBC) S-specific IgG (%S IgG) responses were assessed using an ELISPOT assay.

**Results:**

The median age of the 37 participants at enrollment was 70.5 years; 30 (81%) had prior SARS-CoV-2 infection, and 76% received Pfizer-BioNTech and 24% Moderna homologous vaccines. After an observed augmented effect with each spike exposure, a decline in the immune response, including %S IgG MBCs, was observed over time; the percent decline decreased with increasing spike exposures. Participants who developed an infection at least two weeks post-receipt of a vaccine were observed to have lower humoral antibody levels than those who did not develop an infection post-receipt.

**Conclusions:**

These findings suggest that understanding the durability of immune responses in this vulnerable NH population can help inform public health policy regarding the timing of booster vaccinations as new variants display immune escape.

## Introduction

The impact of the coronavirus disease 2019 (COVID-19) pandemic, caused by the severe acute respiratory syndrome coronavirus 2 (SARS-CoV-2), on congregate settings such as nursing homes was disproportionate and devastating. In the United States (US), as of March 3, 2024, 172,147 nursing home residents have died from COVID-19, and 2,014,496 have reported having at least one SARS-CoV-2 infection [1].

As a high-risk setting, nursing home residents and staff were prioritized by the Advisory Committee on Immunization Practices (ACIP) to receive the mRNA COVID-19 vaccines after the issuance of emergency use authorizations (EUA) by the US Food and Drug Administration (FDA) in 2020 [2]. However, nursing home residents were not included in the clinical trials for the COVID-19 vaccines. As vaccination is a key strategy to prevent COVID-19 morbidity and mortality in nursing home residents, there is an ongoing need to characterize the durability of SARS-CoV-2-specific antibody responses following COVID-19 vaccination in this population due to their increased vulnerability compared to the general population and to inform public health policy about the need and timing of booster vaccinations as new variants display immune escape [3, 4]. Previous studies indicate that nursing home residents can produce SARS-CoV-2 antibodies, albeit of different magnitude, depending on their history of infection, COVID-19 vaccination, and immunosenescence [5–9].

The objectives of this longitudinal evaluation were to characterize the post-vaccine kinetics of humoral (e.g., SARS-CoV-2 specific IgG and neutralizing antibodies) and cellular (i.e., memory B-cell) immune responses, as well as the magnitude and duration of these responses, in nursing home residents who had received the primary series of an mRNA COVID-19 vaccine, including those with and without previous SARS-CoV-2 infection. This evaluation also characterized these immune responses after receipt of the first and second monovalent mRNA COVID-19 vaccine booster doses in a subset of participants.

## Methods

### Population and evaluation design

A prospective longitudinal evaluation of two nursing home resident cohorts was implemented at three metro Atlanta, Georgia facilities. The enrollment of the cohorts occurred at different times: cohort 1 began on October 25, 2020, and ended on November 3, 2020; cohort 2 began on March 17, 2021, and ended on May 24, 2021 (S1 Fig). The evaluation concluded in July 2022. Nursing home residents, irrespective of their SARS-CoV-2 infection history or status, were invited to participate if they had the decision-making capacity to provide written consent by self and/or by their legally authorized representative and had received or were receiving the primary series of either the Pfizer-BioNTech (BNT162b2) or Moderna (mRNA-1273) mRNA COVID-19 vaccine. The exclusion criteria included refusing the COVID-19 vaccine, or the inability to undergo phlebotomy.

A detailed questionnaire was completed during the enrollment and follow-up visits, followed by electronic chart abstraction (S1 Text). Participants were interviewed to obtain information on demographics, COVID-19 signs and symptoms, and hospitalizations. Blood specimens and anterior nasal swabs were collected during enrollment and follow-up visits (S1 Text). Sequential blood samples were collected at specific time points, decided a priori. Specimens and clinical information were not collected from participants hospitalized during a visit; however, participants could choose to continue in the evaluation upon return to the nursing home facility. Information from questionnaires and chart abstraction was entered into a Research Electronic Data Capture (REDCap) database hosted at the US Centers for Disease Control and Prevention (CDC).

This activity was reviewed by the CDC and was conducted consistent with applicable federal law and CDC policy [10–14].

### Case definitions

A SARS-CoV-2 infected participant was defined as a participant with infection documented in the electronic health record or confirmed by laboratory testing using real-time reverse-transcriptase polymerase chain reaction (rRT-PCR), point of care BinaxNOW^™^ COVID-19 Ag Card antigen test (BinaxNOW), or seroconversion as indicated by the presence of anti-nucleocapsid antibody (anti-N) IgG titer to SARS-CoV-2 above the cut-off for seropositivity using Meso Scale Discovery (MSD) immunoassay (MSD; Rockville, MD, USA).

A reinfected SARS-CoV-2 participant was defined as a participant with previously documented infection and subsequent documentation of infection during the evaluation in the electronic health record or seroconversion (as indicated by the presence of anti-nucleocapsid antibody (anti-N) IgG titer to SARS-CoV-2 above the cut-off for seropositivity using MSD immunoassay after waning was observed after the first infection). Additional criteria included if the participant was confirmed by laboratory testing (BinaxNOW or a fourfold increase in anti-N IgG titer to SARS-CoV-2 observed in a blood draw at least 90 days after the prior infection).

An infection-naïve participant was defined as having an absence of a documented SARS-CoV-2 infection and negative SARS-CoV-2 laboratory test results, including seronegative for anti-N antibody.

Vaccine-only immunity was defined as the immune protection in infection-naïve individuals who have had one or more doses of an mRNA COVID-19 vaccine and remained infection-naïve after vaccination initiation.

Hybrid immunity was defined as the immune protection in individuals who have had one or more doses of an mRNA COVID-19 vaccine and have evidence of at least one SARS-CoV-2 infection before or after vaccination initiation.

Spike exposure was defined as exposure to the viral spike protein due to either SARS-CoV-2 infection(s) or mRNA COVID-19 vaccine(s) (S1 Table). The primary series of COVID-19 vaccine was considered as two spike exposures.

Participants who passed away during the evaluation period were categorized as deaths.

### Specimen testing

#### Serology testing

Plasma specimens were tested for immunoglobulin G (IgG) antibodies to SARS-CoV-2 nucleocapsid (N) and spike (full-length spike (S) and receptor binding domain (RBD)) protein antigens using a multi-spot electrochemiluminescent immunoassay (V-PLEX SARS-CoV-2 Panel 2 Kit, MSD, Rockville, MD). Tests were performed according to the manufacturer’s instructions, with samples evaluated at 1/5,000 and 1/50,000 dilutions. Specimen IgG concentrations were interpolated from a standard curve and calibrated by the manufacturer to the 1^st^ WHO International Standard for anti-SARS-CoV-2 Ig (NIBSC code 20/136). Results are reported as Binding Antibody Units (BAU/mL). Seropositivity thresholds were defined by the manufacturer and listed in the test kit insert as follows: anti-S IgG 17.66 BAU/mL, anti-RBD IgG 14.64 BAU/mL, and anti-N IgG 11.80 BAU/mL.

The neutralization antibody capacity of plasma was assessed by measuring the inhibition of angiotensin-converting enzyme 2 (ACE2) binding to SARS-CoV-2 wild-type S protein using a competitive electrochemiluminescent surrogate virus neutralization immunoassay (V-PLEX SARS-CoV-2 Panel 2 (ACE2) Kit, MSD, Rockville, MD). Tests were performed according to the kit instructions, with samples evaluated in duplicate at 1/100 dilution. Results are reported as percent spike (%Spike) inhibition using the formula: %Spike Inhibition = 1 –(Average Sample Signal/Average Diluent-only Signal) x 100. The functional antibody response threshold was set at 80% inhibition.

#### Memory B-cell

Peripheral blood mononuclear cells (PBMCs) collected at enrollment, at six months, post-booster, and at the end of the evaluation were used to assess S-specific IgG and IgA memory B-cell (MBC) responses using an MBC antibody-secreting cells (ASC) ELISPOT assay [15]. Whole blood was collected in sodium heparin tubes, and PBMCs were isolated within 24 hours of collection (S1 Text).

### Statistical analysis

Descriptive statistics and longitudinal linear mixed effects modeling were used to separately describe and analyze the trajectories of titers of plasma antibodies (anti-SARS-CoV-2 S IgG, anti-RBD IgG, and anti-N IgG). For these analyses, a participant’s spike exposure status changed when a participant acquired an infection or received a dose of an mRNA vaccine. Geometric mean antibody concentration titers (GMT) of plasma antibodies were calculated for all participants with specimen collection based on their last spike exposure. Arithmetic means were calculated for neutralizing antibodies for the same time periods. Pearson’s correlation coefficient was used to evaluate the relationship between anti-S IgG titers and neutralizing antibodies. A similar analysis was performed between percent S IgG (%S IgG) MBCs and anti-S IgG as well as %Spike inhibition and %S IgG MBC. A *p* <0.05 was considered statistically significant. All statistical analyses were performed using R Studio, version 2022.07.0, build 548 (R Group for Statistical Computing).

We performed modeling of the kinetics of log-transformed antibody titers and neutralizing antibodies through a linear mixed-effect model starting at the peak level for each participant from the time of their last spike exposure. Each mixed effect model included the fixed effect comprising the time from the last spike exposure and exposure group and random effects for participants and time. Interaction effects were tested using likelihood ratio testing that compared models with and without the interaction. Interactions analyzed include the interaction between time of exposure and exposure group, between time of exposure and sex, between time of exposure and type of vaccine, between time of exposure and race, and between time of exposure and immunocompromised status (S1 Text). Bonferroni-adjustment of alpha value was made for multiple comparisons in linear mixed effect models to avoid inflation of the type I error; an adjusted *p*-value of <0.006 was considered statistically significant.

To evaluate post-vaccination infections (≥14 days post-receipt of a vaccine dose), observations of mean antibody titer values from these participants were compared with observations from participants that did not develop a post-vaccination infection by performing a t-test; a *p*<0.05 was considered statistically significant. Matching was done on the type of exposure and serology collected at a similar time point within a 7-day window.

Participant data were analyzed according to their cumulative number of spike exposures, i.e., SARS-CoV-2 infection and mRNA vaccine at the time of sample collection. During this evaluation, four participants with hybrid immunity were reclassified to re-infection based on anti-N antibody. Calibration of the SARS-CoV-2 antibody assays to the 1^st^ WHO international standard for anti-SARS-CoV-2 IgG allowed us to visually assess antibody concentrations in our evaluation cohort to those published: (1) computed average overall protective threshold of 154 BAU/mL for wild type, 95% Pfizer BNT162b2 vaccine effectiveness (VE) against COVID-19 (for two doses against wild type: 530 anti-S IgG BAU/mL; [16]) and (2) 90% Moderna mRNA-1273 VE against COVID-19 (for two doses against wild type: 298 anti-S IgG BAU/mL and 775 anti-RBD IgG BAU/mL; [17]) (S1 Dataset).

## Results

### Participant characteristics

**Fig 1:**
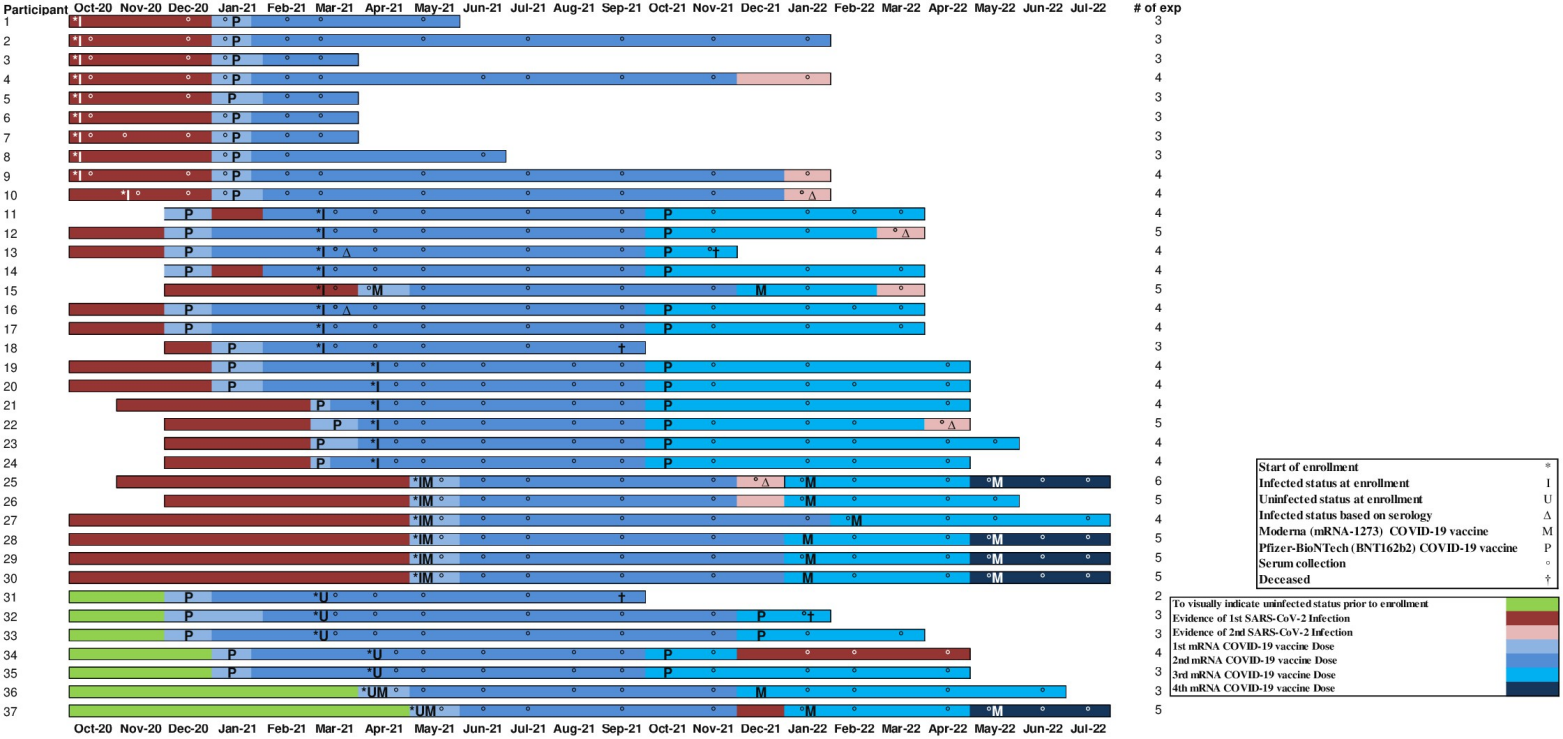
Participant timeline from enrollment until the end of the evaluation period in nursing home cohorts—Georgia, December 2020–July 2022 (n = 37). The various colors represent time from one spike exposure to the next. Spike exposure was defined as exposure to the viral spike protein due to either SARS-CoV-2 infection(s) or mRNA COVID-19 vaccine(s) (S1 Table). The primary series of COVID-19 vaccine was considered as two spike exposures. A SARS-CoV-2 infected participant was defined as a participant with infection documented in the electronic health record or confirmed by laboratory testing using real-time reverse-transcriptase polymerase chain reaction, point of care BinaxNOW^™^ COVID-19 Ag Card antigen test, or seroconversion as indicated by the presence of anti-nucleocapsid antibody (anti-N) IgG titer to SARS-CoV-2 above the cut-off for seropositivity using MSD immunoassay. A reinfected SARS-CoV-2 participant was defined as a participant with previously documented infection and subsequent documentation of infection during the evaluation in the electronic health record or seroconversion (as indicated by the presence of anti-nucleocapsid antibody (anti-N) IgG titer to SARS-CoV-2 above the cut-off for seropositivity using MSD immunoassay after waning was observed after the first infection). Additional criteria included if the participant was confirmed by laboratory testing (BinaxNOW or a fourfold increase in anti-N IgG titer to SARS-CoV-2 observed in a blood draw at least 90 days after the prior infection). The evaluation period covered the wild type [March 2020], alpha (B.1.1.7) [December 2020], Delta (B.1.617.2) [April 2021], and Omicron (B.1.1.529) [November 2021] waves based on detection in Georgia. Participant status at completion:
5 participants passed away during the evaluation (Participants #3, #13, #18, #31, #32); 2 with pneumonia of unknown etiology, none attributed to COVID-19, 1 with cancer, 1 with decline in health, and lastly, 1 with complications from a urinary traction infection.5 participants (Participants #1, #5–8) decided to stop participating partway through the evaluation.27 (Participants #2, #4, #9–12, #14–17, #19–#30, #34–37) participants completed their time in the evaluation.

The results presented here are for all 37 participants across the two cohorts, with the timeline for each participant presented in Fig 1, including but not limited to new infections, re-infections, and mRNA COVID-19 vaccine receipt. Based on spike exposures, each participant’s longitudinal humoral antibody responses are shown in (S2A Fig *(Anti-Spike IgG)*, S2B Fig *(Anti-RBD IgG)*, S2C Fig *(Anti-N IgG)*, S2D Fig *(Neutralizing capacity)*).

At enrollment, the 37 participants had been residing in their respective nursing home facility for a median of 20.8 months (Q1–Q3 11.9–38.6 months). The median age was 70.5 years (Q1–Q3 64.9–78.6 years); 20 (54%) were female, 21 (57%) of the participants identified as White non-Hispanic, and 29 (78%) had at least three existing co-morbid conditions (Table 1). At enrollment, 30 (81%) had evidence of a prior history of SARS-CoV-2 infection, and 7 (19%) had no evidence of SARS-CoV-2 infection.

**Table 1:** Demographic characteristics of nursing home residents by infection status and mRNA COVID-19 vaccine type at enrollment—Georgia, December 2020–July 2022, n = 37.

Characteristics	SARS-CoV-2 infected at enrollment *			SARS-CoV-2 infection-naive at enrollment ^†^			Overall
	Pfizer (n = 23)	Moderna (n = 7)	Total (n = 30)	Pfizer (n = 5)	Moderna (n = 2)	Total (n = 7)	Total (n = 37)
	n %	n %	n %	n %	n %	n %	n %
**Age (median, Q1–Q3), years**	71.7	65.3	70.5	74	72.7	74	70.5
	(64.9–78.6)	(62.5–77.0)	(64.9–77.7)	(64.2–87.0)	(66.8–78.6)	(64.2–87.0)	(64.9–78.6)
**Sex**							
Male	10 (43)	5 (71)	15 (50)	1 (20)	1 (50)	2 (29)	17 (46)
Female	13 (57)	2 (29)	15 (50)	4 (80)	1 (50)	5 (71)	20 (54)
**Race**							
White	17 (74)	3 (43)	20 (67)	4 (80)	0 (0)	4 (57)	24 (65)
Black	6 (26)	4 (57)	10 (33)	1 (20)	2 (100)	3 (43)	13 (35)
**Ethnicity**							
Non-Hispanic	20 (87)	7 (100)	27 (90)	4 (80)	2 (100)	6 (86)	34 (92)
Hispanic	1 (4)	0	1 (3)	0	0	0	1 (3)
Not Specified	2 (9)	0	2 (7)	1 (20)	0	1 (14)	2 (5)
**Duration of stay in the facility (median, Q1–Q3), months**	20.8	21.5	21.2	15.4	69.3	15.4	20.8
	(10.7–41.1)	(13.1–52.4)	(10.8–41.1)	(13.7–25.4)	(0.8–137.8)	(13.3–30.1)	(11.9–38.6)
**Days between date of COVID-19 diagnosis to first mRNA COVID-19 vaccine dose, median (Q1–Q3)**	90	202	105				
	(80–105)	(139–209)	(84–196)				
**Days between first and second doses of mRNA COVID-19 vaccine (median, Q1–Q3)**	21 (21–22)	31 (31–31)	21 (21–31)	23 (23–31)	30 (28–31)	28 (23–31)	22 (21–31)
***Underlying condition*** ^‡^							
≥3 underlying conditions	18 (78)	4 (57)	22 (73)	5 (100)	2 (100)	7 (100)	29 (78)
Hypertension	19 (83)	4 (57)	23 (77)	5 (100)	2 (100)	7 (100)	30 (81)
Cerebrovascular accident	9 (39)	3 (43)	12 (40)	2 (40)	2 (100)	4 (57)	16 (43)
Heart failure	8 (35)	1 (14)	9 (30)	1 (20)	2 (100)	3 (43)	12 (32)
Coronary artery disease	8 (35)	1 (14)	9 (30)	2 (40)	0 (0)	2 (29)	11 (30)
Neurologic disease	11 (48)	5 (71)	16 (53)	2 (40)	2 (100)	4 (57)	20 (54)
Diabetes mellitus	8 (35)	1 (14)	9 (30)	2 (40)	0	2 (29)	11 (30)
Chronic kidney disease	6 (26)	1 (14)	7 (23)	1 (20)	0	1 (14)	8 (22)
Asthma	2 (9)	0	2 (7)	0	1 (50)	1 (14)	3 (8)
Cancer	3 (13)	0	0	0	0	0	3 (8)
Chronic obstructive pulmonary disease (non-asthma)	3 (13)	0	3 (10)	0	0	0	3 (8)
Chronic obstructive pulmonary disease (non-asthma)	3 (13)	0	3 (10)	0	0	0	3 (8)
							0
**Former/current smoker**	14 (61)	5 (72)	19 (64)	2 (40)	1 (50)	3 (43)	22 (60)

*A SARS-CoV-2 infected participant was defined at enrollment as a participant with infection documented in the electronic health record or confirmed by laboratory testing using real-time reverse-transcriptase polymerase chain reaction (rRT-PCR) or point of care BinaxNOW^™^ COVID-19 Ag Card antigen test (BinaxNOW).

^†^An infection-naïve participant was defined as having an absence of a documented SARS-CoV-2 infection and negative SARS-CoV-2 laboratory test results, including seronegative for anti-N antibody.

^‡^Underlying conditions are presented in descending order based on the ‘Overall’ column.

Overall, 28 (76%) received the Pfizer-BioNTech, and 9 (24%) received the Moderna mRNA COVID-19 vaccines; all received the homologous mRNA COVID-19 vaccines with 3- or 4-week intervals between the first and second doses per the manufacturer’s recommendation. The monovalent booster doses were provided as per the various ACIP recommendations. None of the participants received heterologous mRNA vaccines during this evaluation period.

Censoring occurred due to participant death (n = 5; 2 due to pneumonia of unknown etiology, neither of which was attributed to COVID-19, 1 due to cancer, 1 due to decline in health status, and 1 due to complications from a urinary tract infection) and participants declining to continue with evaluation (n = 5).

### Serological results

#### Binding antibodies

**Fig 2:**
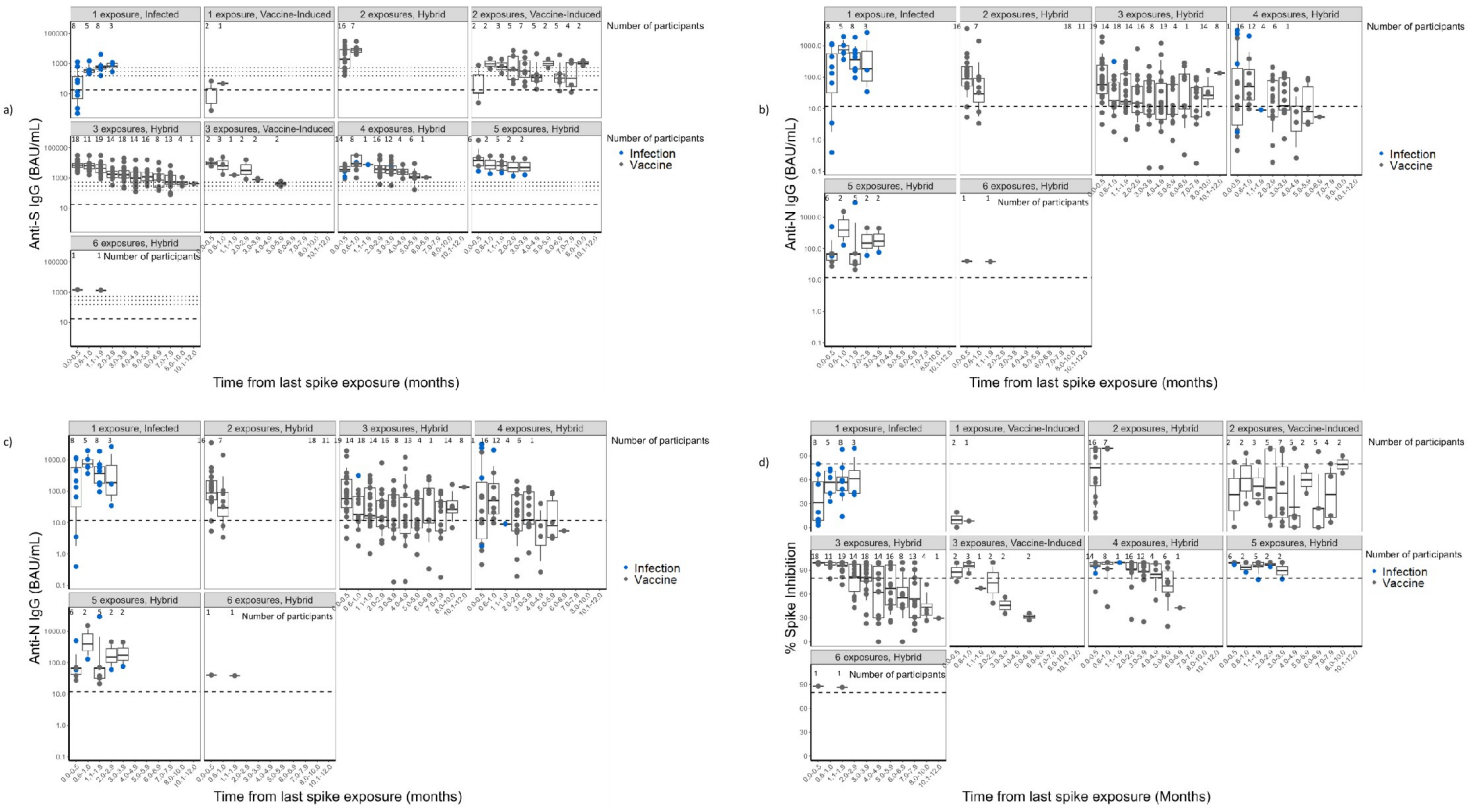
Distribution of IgG antibodies to SARS-CoV-2 spike (full-length spike, S, and S1 subunit receptor binding domain, RBD) and nucleocapsid (N) in nursing home residents—Georgia, December 2020–July 2022 (n = 37). **A**: Distribution of anti-SARS-CoV-2 spike (S) IgG antibodies. Footnotes: N: number of participants at each time point; anti-S IgG: anti-SARS-CoV-2 Spike IgG; BAU/mL: Binding antibody units/mL. Y-axis: Antibody levels in BAU/mL in logarithmic scale. This graph shows the geometric mean titers of measured anti-S IgG antibodies. Blue dots (SARS-CoV-2 infection) and black dots (mRNA COVID-19 vaccine) represent the last exposure type. Seropositivity thresholds were defined by the manufacturer and listed in the kit insert as follows: anti-S IgG 17.66 BAU/mL, as indicated by the lower dashed line. Calibration of the SARS-CoV-2 antibody assays to the 1^st^ WHO international standard for anti-SARS-CoV-2 IgG allowed for visual assessment of antibody concentrations in our evaluation to those associated with a computed average overall protective threshold of 154 BAU/mL for wild type, 95% Pfizer BNT162b2 VE against COVID-19 (for two doses against wild type 530 anti-S IgG BAU/mL; Goldblatt, 2022) and 90% Moderna mRNA-1273 VE against COVID-19 (for two doses against wild type, 298 anti-S IgG BAU/mL and 775 anti-RBD IgG BAU/mL; Gilbert 2022)—as indicated by the three upper dotted lines. **B**: Distribution of anti-SARS-CoV-2 Receptor Binding Domain (RBD) IgG antibodies. Footnotes: N: number of participants at each time point; anti-RBD IgG: anti-SARS-CoV-2 Receptor-Binding Domain IgG; BAU/mL: Binding antibody units/mL. Y-axis: Antibody levels in BAU/mL in logarithmic scale. This graph shows the geometric mean titers of measured anti-RBD IgG antibodies. The last exposure type is represented by a blue dot (SARS-CoV-2 infection) and a black dot (mRNA COVID-19 vaccine). Seropositivity thresholds were defined by the manufacturer and listed in the kit insert as follows: anti-RBD IgG 14.64 BAU/mL as indicated by the lower dashed line. Calibration of the SARS-CoV-2 antibody assays to the WHO 1^st^ international standard for anti-SARS-CoV-2 immunoglobulin allowed visual assessment of antibody concentrations in our evaluation to those associated with 90% Moderna mRNA-1273 VE against COVID-19 (775 anti-RBD IgG BAU/mL—as indicated by the upper dotted line; Gilbert, 2022). **C**: Distribution of anti-SARS-CoV-2 Nucleocapsid (N) IgG antibodies among those with hybrid immunity. Footnotes: anti-N IgG: anti-SARS-CoV-2 Nucleocapsid IgG; BAU/mL: Binding antibody units/mL. Y-axis: Antibody levels in BAU/mL in logarithmic scale. This graph shows the geometric mean titers of measured anti-N IgG antibodies. The last exposure type is represented by a blue dot (SARS-CoV-2 infection) and a black dot (mRNA COVID-19 vaccine). Seropositivity thresholds were defined by the manufacturer and listed in the kit insert as follows: anti-N IgG 11.80 BAU/mL, as indicated by the dashed line. Although the vaccine exposure has been included as part of the spike exposures, the anti-N IgG is only affected by a SARS-CoV-2 infection. **D**: Virus Neutralizing Capacity using percent spike inhibition. Footnotes: Y-axis: Percent spike inhibition. This graph shows the percent spike inhibition (virus neutralizing capacity). The last exposure type is represented by a blue dot (SARS-CoV-2 infection) and a black dot (mRNA COVID-19 vaccine). The functional antibody response threshold was set at 80% inhibition, as indicated by the dashed line.

All participants showed detectable anti-S IgG (Fig 2A). In general, a boosting effect was seen with each spike exposure (i.e., an mRNA COVID-19 vaccine and/or a new or a second SARS-CoV-2 infection) (S2 Table), followed by waning. Anti-RBD IgG followed the same pattern as the anti-S IgG antibody levels (Fig 2B). Among those with hybrid immunity, the anti-N IgG antibodies rose during periods of reinfection, with subsequent declines observed over time (Fig 2C).

The longest observation period was for participants with three spike exposures (approximately 6 months) (Fig 2A). As per Table 2, the 3-exposure vaccine-only participants (mRNA COVID-19 vaccine primary series with recent receipt of a first booster) elicited an anti-S IgG peak response of 9394.7 BAU/mL and a decline of 31.2% decline per month while the 3-exposure hybrid immunity participants (infection on or before enrollment followed by mRNA COVID-19 vaccine primary series) elicited a anti-S IgG peak 5487.7 BAU/mL with 22.6% decline per month; no statistical difference was observed in the percentage decline (p = 0.12, adjusted p-values of <0.006 were considered statistically significant). No statistical difference was observed in the percentage decline of anti-S IgG antibodies between the mRNA COVID-19 vaccines among those with hybrid immunity with three spike exposures (p = 0.46) (Table 2).

**Table 2:** Linear mixed effects modeling parameter estimates of IgG antibodies to SARS-CoV-2 spike (full length spike, S) and neutralizing antibodies in nursing home residents—Georgia, December 2020– July 2022.

Groups	Anti-Spike IgG							Neutralizing Antibodies						
	No. of observations	No. of participants	Estimate (log_10_)	Standard Error	likelihood ratio p-value*	peak (BAU/ml)	% decline/month	No. of observations	No. of participants	Estimate (log_10_)	Standard Error	likelihood ratio p-value*	peak (% spike)	slope (%/month)
**2 spike exposures** †														
Hybrid Immunity‡	23	17	-0.39	0.11	0.02	3470.90	-59.08	23	17	5.70	6.90	0.16	74.77	5.70
Vaccine-induced^§^	44	7	0.29	0.11		857.96	-19.58	41	7	-9.87	7.04		58.66	-4.17
**3 spike exposures** †														
Hybrid Immunity	154	31	-0.11	0.01	0.12	5487.64	-22.64	160	31	-6.18	1.02	0.42	99.17	-6.18
Vaccine-induced	12	5	-0.05	0.03		9394.72	-31.24	12	5	-2.77	3.43		95.21	-8.95
**Hybrid Immunity** ‡ **: 3 vs. 4 spike exposures** †														
3 exposures	154	31	-0.11	0.01	0.82	5489.91	-22.54	160	31	-6.17	0.92	0.06	99.17	-6.17
4 exposures	60	24	0.00	0.01		6292.06	-23.13	60	24	2.94	1.58		96.63	-3.23
**Hybrid Immunity** ‡ **: 4 spike exposures versus 5 Spike exposures** †														
4 exposures	60	24	-0.11	0.01	0.39	6308.83	-22.71	60	24	-3.62	0.93	0.57	96.83	-3.62
5 exposures	17	9	-0.04	0.04		11519.51	-29.05	16	9	1.37	2.45		98.36	-2.25
**Hybrid Immunity**‡ **with 3 Spike Exposures**†														
By Sex														
Females	82	15	-0.11	0.01	0.91	4043.25	-22.43	83	15	-6.42	1.45	0.80	95.85	-6.42
Males	72	16	0.00	0.02		7298.42	-22.73	77	16	0.51	2.05		102.19	-5.91
By mRNA COVID-19 Vaccine														
Moderna	32	8	-0.12	0.02	0.46	11296.63	-24.27	38	8	-4.28	1.92	0.24	100.58	-4.28
Pfizer	122	23	0.01	0.02		4271.96	-21.93	122	23	-2.62	2.24		98.70	-6.90
By Race														
Black	50	11	-0.10	0.01	0.13	10708.88	-19.80	51	11	-3.92	1.74	0.12	103.26	-3.92
White	104	20	-0.02	0.02		3803.69	-24.15	109	20	-3.30	2.10		96.89	-7.22
By Immunocompromised Status^¶^														
No	79	14	-0.10	0.01	0.20	4410.80	-20.96	79	14	-6.53	1.49	0.75	98.08	-6.53
Yes	75	17	-0.02	0.01		6710.36	-24.40	81	17	0.64	2.05		100.05	-5.89

*p-values of <0.006 were considered statistically significant

^†^Spike exposure is defined as exposure to the viral spike protein due to either a SARS-CoV-2 infection(s) or a dose(s) of an mRNA COVID-19 vaccine receipt. The primary series was considered as two spike exposures. Please refer to S1 Table for further explanation

^‡^Hybrid immunity was defined as the immune protection in individuals who have had one or more doses of an mRNA COVID-19 vaccine and have evidence of at least one SARS-CoV-2 infection before or after vaccination initiation.

^§^Vaccine-induced immunity was defined as the immune protection in individuals who have had one or more doses of an mRNA COVID-19 vaccine and remained infection-naive before or after vaccination initiation.

^¶^A moderate or severely immunocompromising condition included the following: recent or active malignancy, bone marrow transplant, solid organ transplant, primary or secondary immune deficiency, or the use of oral or intravenous steroids for more than a month or any immunosuppressant drugs

**Fig 3:**
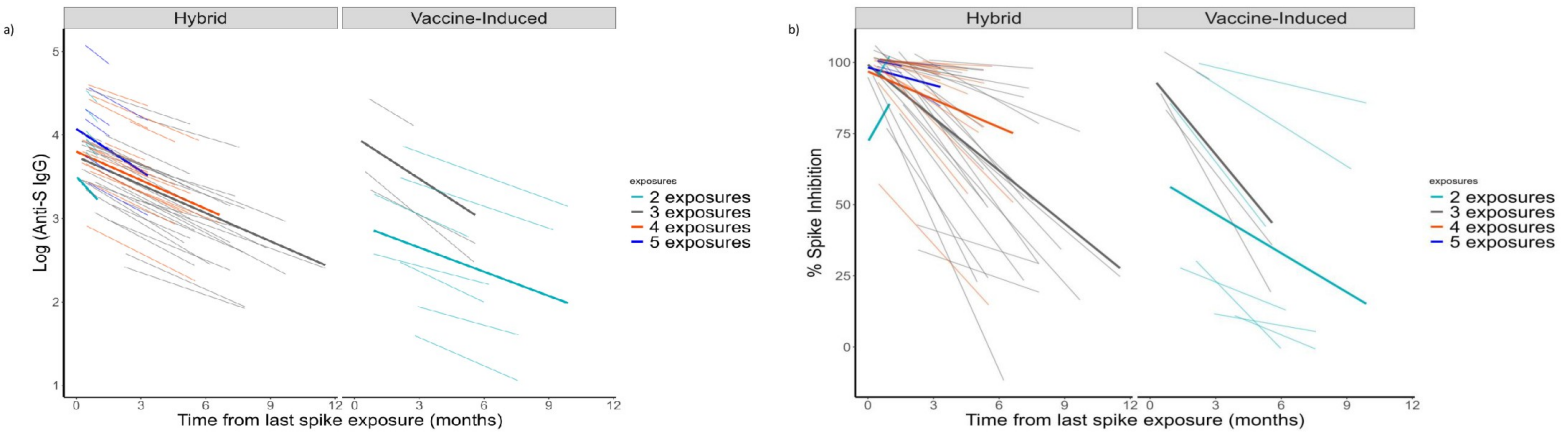
Linear mixed effect model comparing spike exposure groups in a nursing home cohort—Georgia, December 2020–July 2022, n = 37. **A**: Linear Mixed Effect Modeling for anti-SARS-CoV-2 Spike (S) IgG. Footnote: We explored modeling the kinetics of log-transformed antibody titers through a linear mixed-effect model starting at the peak level for each participant from the time of their last spike exposure. Each mixed effect model included the fixed effect comprising the time from the last spike exposure and exposure group and random effects for participants and time. Bonferroni-adjustment of alpha value was made for multiple comparisons in linear mixed effect models to avoid inflation of the type I error; adjusted *p*-value of <0.006 were considered statistically significant. Light-colored lines indicate individual participant responses; Dark-colored lines indicate mean estimates for the spike exposure groups. **B**: Linear mixed effect model for percent spike inhibition (virus neutralizing capacity). Footnotes: We explored modeling the kinetics of log-transformed neutralizing antibody titers through a linear mixed effect model starting at the peak level for each participant from the time of their last spike exposure. Each mixed effect model included the fixed effect comprising the time from the last spike exposure and exposure group and random effects for participants and time. Interaction effects were tested using likelihood ratio testing that compared models with and without the interaction. Bonferroni-adjustment of alpha value was made for multiple comparisons in linear mixed effect models to avoid inflation of the type I error; adjusted *p*-value of <0.006 were considered statistically significant. Light-colored lines indicate individual participant responses; Dark-colored lines indicate mean estimates for the spike exposure groups.

Among those with hybrid immunity with 4 and 5 spike exposures, the anti-S IgG antibodies increased after each exposure but steadily declined over time (Fig 3A). When comparing increasing exposures among those with hybrid immunity, an additional exposure elicited a higher peak immune response than the previous exposure; however, the percentage decline over time remained similar and was not significantly different (Table 2).

#### Neutralizing antibodies (antibodies with ACE2 binding inhibition activity)

Participants showed variable surrogate neutralizing antibody responses, whether to vaccination or infection. Surrogate neutralization capacity (i.e., %spike inhibition >80% threshold) was maintained over more extended periods of time as the number of spike exposures increased (Fig 2D). In hybrid and vaccine-only participants, the virus neutralization decay was observed after each spike exposure, but with no significant differences in percentage decline (Fig 3B, Table 2).

**Fig 4:**
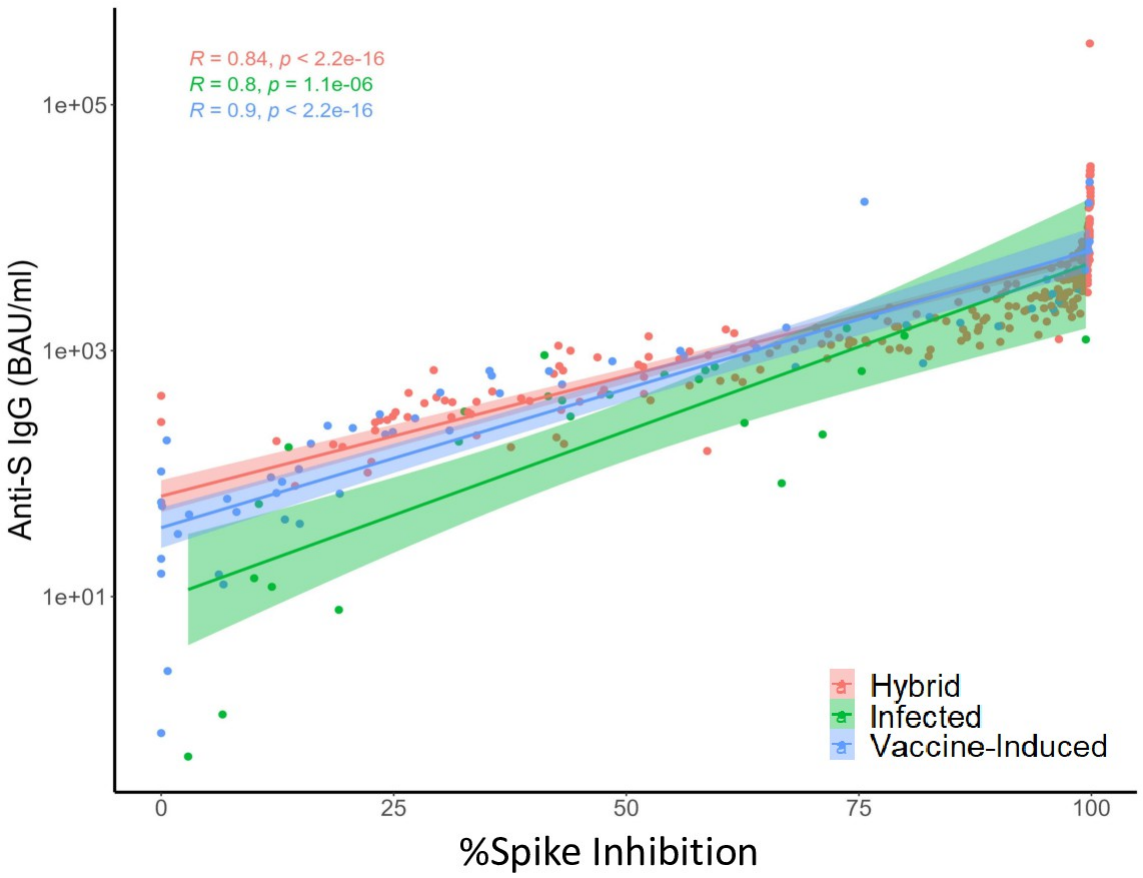
Relationship between anti-SARS-CoV-2 Spike IgG and percent spike inhibition in nursing home residents—Georgia, December 2020–July 2022, n = 37. Pearson’s correlation coefficient^®^ was used to evaluate the relationship between anti-S IgG titers and percent spike inhibition. A SARS-CoV-2 infected participant was defined as a participant with infection documented in the electronic health record or confirmed by laboratory testing using real-time reverse-transcriptase polymerase chain reaction (rRT-PCR), point of care BinaxNOW^™^ COVID-19 Ag Card antigen test (BinaxNOW), or seroconversion as indicated by the presence of anti-nucleocapsid antibody (anti-N) IgG titer to SARS-CoV-2 above the cut-off for seropositivity using Meso Scale Discovery (MSD) immunoassay (MSD; Rockville, MD, USA). Vaccine-only immunity was defined as the immune protection in infection-naïve individuals who have had one or more doses of an mRNA COVID-19 vaccine and remained infection-naïve after vaccination initiation. Hybrid immunity was defined as the immune protection in individuals who have had one or more doses of an mRNA COVID-19 vaccine and have evidence of at least one SARS-CoV-2 infection before or after vaccination initiation.

In the hybrid immunity and vaccine-only immunity participants, a strong positive correlation was observed between virus neutralization and anti-S IgG titers (hybrid immunity: R = 0.84; vaccine-only immunity: R = 0.9) (Fig 4).

**Fig 5:**
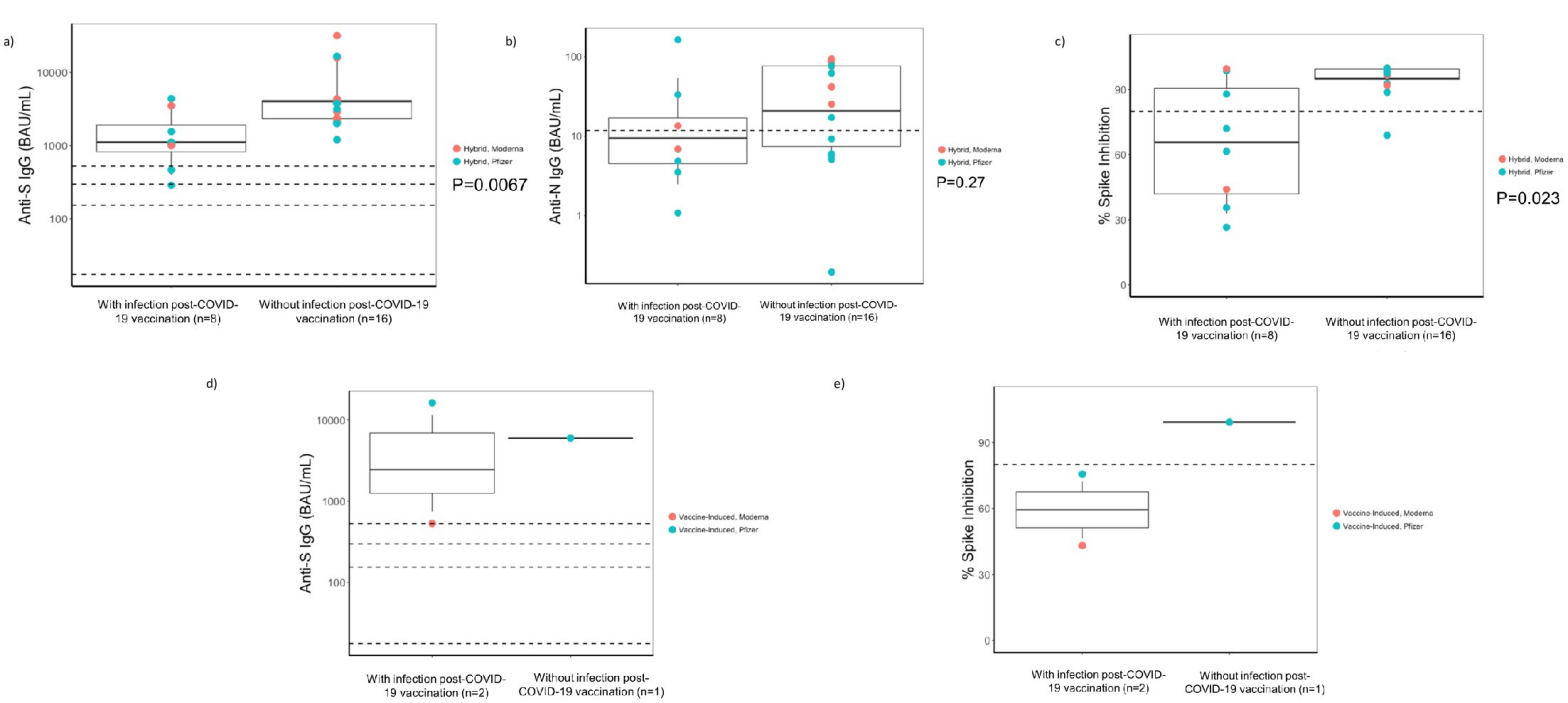
Immune responses in nursing home participants with and without infection post-COVID-19 vaccination—Georgia, December 2020–July 2022. **A**: Distribution of anti-SARS-CoV-2 Spike (S) IgG among the hybrid immunity in nursing home residents with and without infection post-COVID-19 vaccination (n = 24). anti-S IgG: anti-SARS-CoV-2 Spike IgG; BAU/mL: Binding antibody units/mL. Footnotes: Y-axis: Antibody levels in BAU/mL in logarithmic scale. To evaluate infections post-COVID-19 vaccination, observations of mean antibody titer values from participants who developed infection ≥14 days postvaccination were compared with observations from participants who did not develop an infection post-COVID-19 vaccination by performing a t-test; a p-value of <0.05 was considered statistically significant. Matching was done on the type of exposure and serology collected at a similar time point within a 7-day window. The p = 0.0067 was statistically significant. **B**: Distribution of anti-SARS-CoV-2 Nucleocapsid (N) IgG antibodies among the hybrid immunity in nursing home residents with and without infections post-COVID-19 vaccination (n = 24). anti-N IgG: anti-SARS-CoV-2 Nucleocapsid IgG; BAU/mL: Binding antibody units/mL. Y-axis: Antibody levels in BAU/mL in logarithmic scale. Footnote: To evaluate infections post-COVID-19 vaccination, observations of mean antibody titer values from participants who developed infection ≥14 days postvaccination were compared with observations from participants who did not develop an infection post-COVID-19 vaccination by performing a t-test; a p-value of <0.05 was considered statistically significant. Matching was done on the type of exposure and serology collected at a similar time point within a 7-day window. The p = 0.27 was not statistically significant. **C**: Virus neutralizing capacity among the hybrid immunity in nursing home residents with and without infections post-COVID-19 vaccination (n = 24). Virus neutralizing capacity = percent spike inhibition; Y axis in %. Footnote: To evaluate infections post-COVID-19 vaccination, observations of mean antibody titer values from participants who developed infection ≥14 days postvaccination were compared with observations from participants who did not develop an infection post-COVID-19 vaccination by performing a t-test; a p-value of <0.05 was considered statistically significant. Matching was done on the type of exposure and serology collected at a similar time point within a 7-day window. The p = 0.023 was not significant. **D**: Distribution of anti-SARS-CoV-2 Spike (S) IgG among the vaccine-induced immunity in nursing home residents with and without infections post-COVID-19 vaccination (n = 3). Footnote: anti-S IgG: anti-SARS-CoV-2 Spike IgG; BAU/mL: Binding antibody units/mL. Y-axis: Antibody levels in BAU/mL in logarithmic scale. To evaluate infections post-COVID-19 vaccination, observations of mean antibody titer values from participants who developed infection ≥14 days postvaccination were compared with observations from participants who did not develop an infection post-COVID-19 vaccination by performing a t-test; a p-value of <0.05 was considered statistically significant. Matching was done on the type of exposure and serology collected at a similar time point within a 7-day window. We were unable to assess statistically due to small numbers; hence, no p- values are provided. **E**: Virus neutralizing capacity among the vaccine-induced residents, comparing those with and without infection post-COVID-19 vaccination (n = 3). Footnote: Virus neutralizing capacity = percent spike inhibition; Y axis in %. To evaluate infections post-COVID-19 vaccination, observations of mean antibody titer values from participants who developed infection ≥14 days postvaccination were compared with observations from participants who did not develop a post-COVID-19 vaccination infection by performing a t-test; a p-value of <0.05 was considered statistically significant. Matching was done on the type of exposure and serology collected at a similar time point within a 7-day window. We were unable to assess statistically due to small numbers; hence, no p- values are provided.

Among participants with hybrid immunity who received a booster followed by reinfection, the anti-S GMT (p = 0.0067, Fig 5A) and virus neutralization capacity were significantly lower (p = 0.023, Fig 5C) within 7 days of reinfection than those who did not develop reinfection; anti-N antibodies were not significantly different (p = 0.27, Fig 5B). Similar analyses were performed among those with vaccine-only immunity (Fig 5D and 5E); a statistical outcome could not be ascertained due to fewer observations.

#### Cellular response based on spike exposures: Memory B cells

**Fig 6:**
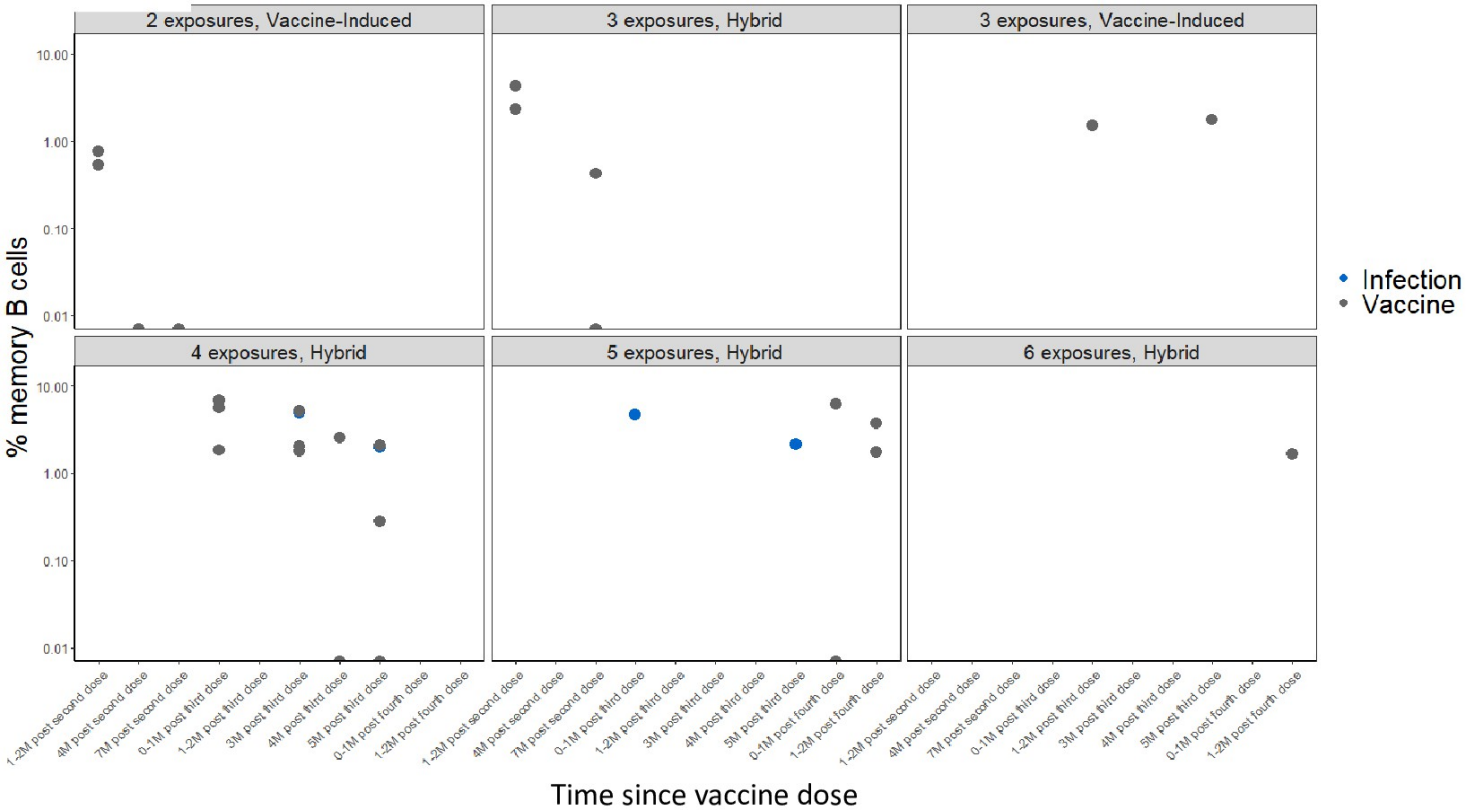
Distribution of full-length Spike IgG memory B cells (MBC) by spike exposure and time since an MRNA COVID-19 vaccine dose in a subset of vaccinated nursing home residents—Georgia, December 2020–July 2022, n = 37. X-axis: Time since an mRNA COVID-19 vaccine dose. Y-axis: Percent (%) Spike IgG Memory B Cells (MBC).

Whole blood was drawn from a subset (n = 15) of participants; at the first draw, there were 12 participants with hybrid- and 3 with vaccine-only-induced immunity. Overall, among participants with hybrid immunity, the % S-specific IgG MBCs increased immediately after receipt of a vaccine dose or a re-infection, with a gradual decline over six months. In participants with vaccine-only immunity, the % S-specific IgG MBCs also increased after receipt of a vaccine dose (Table 3, Fig 6); a decline was observed at four months post-second dose. Statistical comparisons could not be completed due to small sample sizes. S3A–S3C Fig show the % S-specific IgA and nucleocapsid-specific IgG and IgA responses (S3 Table).

**Table 3:** Range of SARS-CoV-2% memory B cells among participants with hybrid and vaccine-induce immunity and number of spike exposure* in nursing home residents–Georgia, December 2020–July 2022, n = 15.

Group	Time Period from the last mRNA COVID-19 vaccine dose (months)	Number of participants	% S IgG MBC (min-max)	%N-IgG	%S IgA	%N IgA	Total IgG
			Min-Max	Min-Max	Min-Max	Min-Max	Min-Max
**Hybrid** ^†^							
3 spike exposures	1–2 months post second dose	2	2.31–4.29	0.00–0.10	0.13–0.32	0.00–0.45	240.7–900.0
3 spike exposures	7 months post second dose	2	0.00–0.42	0.00–0.00	0.00–0.00	0.00–0.00	536.4–622.2
4 spike exposures	1 month post third dose	3	1.85–6.86	0.00–0.31	0.00–6.70	0.00–0.11	1636.4–10607.1
4 spike exposures	3 months post third dose	4	1.79–5.21	0.00–0.00	0.00–1.32	0.00–0.00	1015.6–7615.4
4 spike exposures	4 months post third dose	2	0.00–2.6	0.00–0.00	0.00–0.00	0.00–0.00	116.7–1800.0
4 spike exposures	5 months post third dose	4	0.00–2.0	0.00–0.2	0.00–0.7	0.00–0.00	697.7–1800.0
5 spike exposures	1 month post third dose	1	4.67	2.28	1.45	0.00	3075.00
5 spike exposures	0–1 months post fourth dose	2	0.00–6.2	0.00–0.9	0.00–0.00	0.00–0.1	846.2–3576.9
5 spike exposures	1–2 months post fourth dose	2	1.7–3.7	0.00–0.9	0.00–10.9	0.00–0.00	2036.4–3393.8
5 spike exposures	5 months post third dose	1	2.20	0.00	0.06	0.00	822.40
6 spike exposures	1–2 months post fourth dose	1	1.70	0.10	0.00	0.00	10909.10
**Vaccine-only** ^‡^							
2 spike exposures	1–2 months post second dose	2	0.53–0.76	0.00–0.06	0.00–0.07	0.00–0.16	631.6–2062.5
2 spike exposures	4 months post second dose	1	0.00	0.00	0.22	1.58	2769.20
2 spike exposures	7 months post second dose	1	0.00	0.05	0.00	0.00	4846.20
3 spike exposures	1 month post third dose	1	1.50	0.00	0.00	0.19	3784.10
3 spike exposures	5 months post third dose	1	1.80	0.10	0.50	0.10	3300.00

S-Full length Spike; N-Nucleocapsid

*Spike exposure is defined as exposure to the viral spike protein due to either a SARS-CoV-2 infection(s) or a dose(s) of an mRNA COVID-19 vaccine receipt. The primary series was considered as two spike exposures.

^†^Hybrid immunity was defined as the immune protection in individuals who have had one or more doses of an mRNA COVID-19 vaccine and have evidence of at least one SARS-CoV-2 infection before or after vaccination initiation.

^‡^Vaccine-induced immunity was defined as the immune protection in individuals who have had one or more doses of an mRNA COVID-19 vaccine and remained infection-naive before or after vaccination initiation.

**Fig 7:**
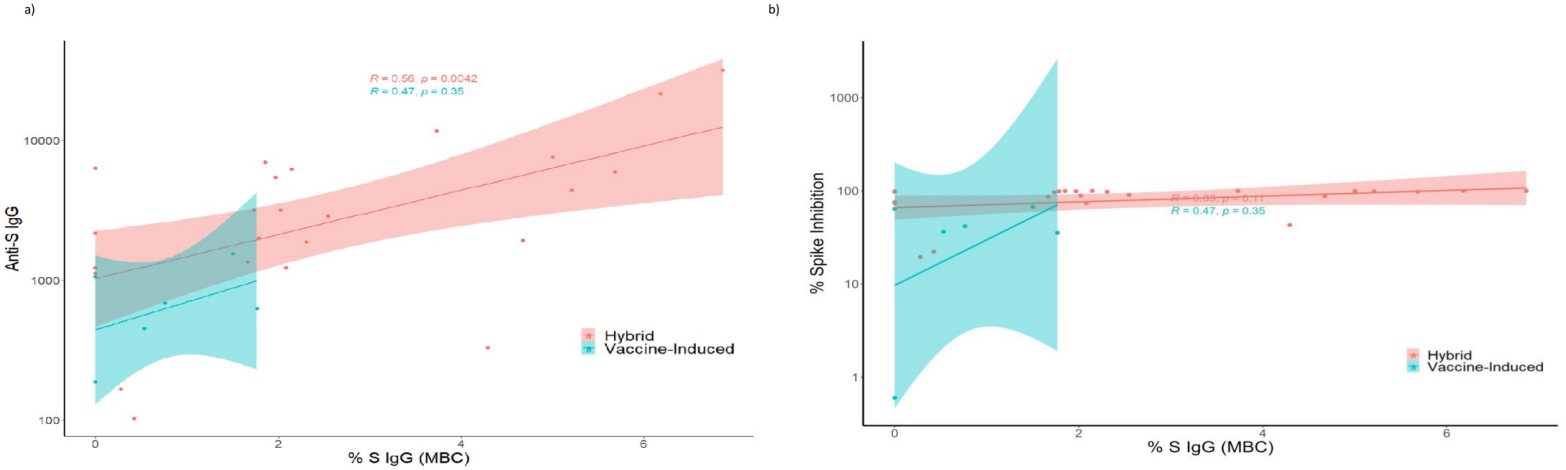
**A**: Relationship between anti-SARS-Cov-2 Spike IgG and Memory B cell S IgG in nursing home residents—Georgia, December 2020–July 2022; n = 15. Pearson’s correlation coefficient (R) was used to evaluate the relationship between anti-S IgG titers and percent spike inhibition (virus neutralizing capacity). Hybrid immunity R = 0.56, p = 0.0042. Vaccine-induced immunity: R = 0.47, p = 0.35. Footnote: Vaccine-only immunity was defined as the immune protection in infection-naïve individuals who have had one or more doses of an mRNA COVID-19 vaccine and remained infection-naïve after vaccination initiation. Hybrid immunity was defined as the immune protection in individuals who have had one or more doses of an mRNA COVID-19 vaccine and have evidence of at least one SARS-CoV-2 infection before or after vaccination initiation. **B**: Relationship between full-length Spike IgG Memory B Cells (MBC) and Percent Spike Inhibition in nursing home residents—Georgia, December 2020–July 2022; n = 15. Pearson’s correlation coefficient (R) was used to evaluate the relationship between full-length Spike IgG Memory B Cells (MBC) and Percent Spike Inhibition. Hybrid immunity R = 0.33, p = 0.11. Vaccine-induced immunity: R = 0.47, p = 0.35. Footnote: Vaccine-only immunity was defined as the immune protection in infection-naïve individuals who have had one or more doses of an mRNA COVID-19 vaccine and remained infection-naïve after vaccination initiation. Hybrid immunity was defined as the immune protection in individuals who have had one or more doses of an mRNA COVID-19 vaccine and have evidence of at least one SARS-CoV-2 infection before or after vaccination initiation.

We observed a moderate, positive correlation between anti-SARS-CoV-2 S IgG and % S-specific IgG MBCs in participants with hybrid immunity (R = 0.56). (Fig 7A). The % S-specific IgG MBCs had a lower positive correlation with virus neutralization (R = 0.33) among those with hybrid immunity (Fig 7B).

## Discussion

Nursing home residents, a population excluded from COVID-19 vaccine clinical trials, are at high risk for COVID-19-associated morbidity and mortality [1, 18, 19], emphasizing the importance of understanding immunity elicited from vaccination and SARS-CoV-2 infection. In this longitudinal evaluation of two convenience cohorts of nursing home residents with 37 participants, we sought to characterize SARS-CoV-2-specific humoral and B-cell responses after infection and mRNA COVID-19 vaccination. Similar to the reports among the general population [20–23], all nursing home participants in these cohorts with hybrid or vaccine-only immunity elicited anti-SARS-CoV-2 antibody response, surrogate neutralizing activity, and MBC responses. The primary series and the monovalent boosters of the mRNA COVID-19 vaccines, Pfizer (BNT1272b2) and Moderna (mRNA-1273), are known to elicit the production of anti-S, anti-RBD, and neutralizing antibodies, in addition to MBC responses in non-nursing home residents [24–26]. While the immune response to both infection and mRNA COVID-19 vaccinations in our evaluation is encouraging, antibody waning was observed in both hybrid immunity and vaccine-only participants after each spike exposure. Antibody declines after each COVID-19 vaccine dose have been described previously in this population and others [9, 27–31]. The decline in post-infection antibody levels has been observed in the population studies performed in the community and healthcare personnel [32–35].

SARS-CoV-2-specific MBCs may be essential for producing neutralizing antibodies [36], long-term protection, helping to prevent reinfection [37, 38], and possibly preventing severe disease with reinfection [39]. We observed a rise in the % S-specific IgG MBCs after each spike exposure, followed by a decline over time, similar to the antibody response. Although there was an insufficient number of time points in our evaluation to assess if the %S IgG MBCs increased over time, as has been observed in the general population [39], the decline in MBCs appeared to be faster in this cohort than in young, healthy adults when compared to a study by Kim et al. where MBC response levels persisted up to nine months [40].

In our evaluation, neutralizing antibodies decay appeared slower among those with hybrid immunity after the 4^th^ and 5^th^ spike exposures when compared to those with ≤3 exposures, although the difference was not statistically significant. Surrogate neutralizing antibodies strongly correlated with anti-S IgG levels and %S IgG MBC among those with hybrid immunity. Among the vaccine-only participants, while the binding antibody anti-S IgG strongly correlated with virus neutralization, only a modest correlation was observed with %S IgG MBC, likely due to the smaller number of available PBMCs for evaluation. These findings indicate that despite the advanced age of these cohorts and medical comorbidities, these nursing home participants elicited functional antibodies and MBCs, which are important for long term protection. However, the threshold for protection is currently undetermined, and the durability of protection in these populations should be monitored to help inform booster recommendations.

The decay in antibodies and MBCs observed in our cohorts of nursing home residents may be attributed to immunosenescence [41]. Published studies have also indicated frailty, defined as a complex phenomenon due to a cumulative decline in multiple physiologic systems resulting in reduced resistance to stressors, as an explanation for decreased post-vaccine antibody response [6, 42, 43], where higher levels of frailty result in decreased SARS-CoV-2 antibody responses, similar to what has been observed following the receipt of other viral vaccines (e.g., Zoster [44]). A study by Shapiro et al. indicated that immune response to a third dose of mRNA COVID-19 vaccine booster dose appears to overcome reduced immunity associated with frailty [45], consistent with our findings, based on linear mixed modeling, indicating reduced decay rates after multiple spike exposures.

Post-vaccination infections have been described in nursing home residents [46, 47]. We observed such infections in our cohorts. Among those hybrid participants who developed reinfection, anti-S and anti-N IgG concentrations and virus neutralization capacity were observed to be lower just before the reinfection when compared to those who did not develop reinfection. Despite such post-vaccination infections, no participants were hospitalized due to SARS-CoV-2 infection, and no participant deaths were attributed to the SARS-CoV-2 infection.

Post-vaccination infections occurring during this evaluation also temporally coincided with the Omicron wave. Previous studies have demonstrated that sera collected after receipt of the primary COVID-19 vaccine series had lower neutralizing activity against the Omicron variant than the original circulating strain of SARS-CoV-2 [48, 49]. Canaday et al., in a prospective longitudinal study, observed that the bivalent COVID-19 mRNA vaccine substantially increased anti-spike IgG and neutralizing antibody titers against Omicron sub-lineages, including BA.1 and BA.4/BA.5, irrespective of previous SARS-CoV-2 infection or previous receipt of 1 or 2 booster doses [27]. However, our evaluation was completed before the availability of the bivalent booster; thus, all vaccinations were ancestral vaccine.

This evaluation has several limitations. First, this was a relatively small convenience sample of residents from 3 nursing home facilities, and therefore, it is difficult to extrapolate the findings to the larger US nursing home resident population. Secondly, participants in these cohorts had different enrollment and vaccination dates and contribution times, which led to differences in where they were in their vaccination timeline and overall days in the evaluation. However, the longitudinal observational design of this evaluation from October 2020 to July 2022 with frequent blood sampling provided detailed information on SARS-CoV-2 antibody dynamics following COVID-19 vaccination and/or infection at a fine-scaled timeline. The specimen collection dates may have affected the tracking of the immune responses, including capturing the accurate peak antibody titers. Thirdly, throughout the evaluation, there were four participants with hybrid immunity who were reclassified based on their serology results (seroconversion by anti-N IgG), making these instances a possible misclassification. Nursing home facility testing results were also used to supplement our data because some of the study visits did not always correspond with the timing of the participant’s illness. Fourthly, due to the evaluation’s observational design, participants moved from the infection naïve to the infected category. Therefore, there were no participants with vaccine-only immunity with more than 3 spike exposures, resulting in a decline in the statistical power of those with vaccine-only, which started small and decreased as time passed. Similarly, we could not generate enough statistical power to demonstrate significance when comparing demographics or co-morbidities for the vaccine-only group as the number of participants with vaccine-only immunity became fewer in number as the evaluation progressed. Linear models only evaluate linear decay, and antibody decay may initially decline and stabilize. We were also unable to analyze the possible factors for the decline in the anti-S IgG and virus neutralization capacity among those who developed reinfection, as all eight participants with reinfections had multiple comorbidities reflective of the cohort. Additionally, the time since vaccination was similar between those who developed reinfection and those who did not. While attempts to ensure timely and adequate PBMC collection were made, there may have been missed opportunities with fewer samples collected. Another limitation is that during the evaluation, we were unable to assess for the existing strain as the assay focused on the wild strain. And lastly, in the linear mixed modeling, the duration of observation time was shorter for participants with higher spike exposures.

In conclusion, these cohorts of nursing home residents were able to elicit immune responses after infection with SARS-CoV-2 and COVID-19 vaccination. However, waning of the immune response over time was observed. Nursing home residents remain vulnerable to severe outcomes of infection due to their comorbidities, immunosenescence, and frailty, the evolution of SARS-CoV-2, and continued community transmission with reduced mitigations. To protect vulnerable populations, continuous longitudinal reassessment of vaccination rates, incidence of SARS-CoV-2 infections, and immune response elicited by SARS-CoV-2 infection and COVID-19 vaccination, in addition to understanding the thresholds for protection, are important to inform data-driven public health infection control strategies, vaccine recommendations, and vaccine development [16, 50]. The findings in this evaluation align with at-the-time vaccine recommendations provided by ACIP for older adults and at-risk populations [2, 51]. Comparing the immune response to vaccination and infection in vulnerable populations with the general population could be helpful to tailor vaccine or prevention strategies. Additionally, understanding the longevity of neutralizing antibodies, the potential roles of T- and B-cells, and close monitoring of viral evolution will be important to the success of vaccination efforts and booster strategies to prevent further morbidity and mortality, especially in vulnerable nursing home populations.

## Supporting information

S1 Text Supplemental methods and results. (DOCX)

S1 Dataset De-identified dataset. (XLSX)

S1 Fig Timeline of evaluation in nursing home residents (N = 37) by cohort (n = 2) and facility (n = 3)—Georgia, October 2020–July 2022. Cohort 1 (n = 10), Cohort 2 (n = 27), and facility (n = 3). Note that for Cohort 1, the intense phase consisted of 4 visits conducted every other day for the first 10 days; for Cohort 2, the intense phase consisted of 4 visits conducted every other week for 2 months. The enrollment period for Cohort 1 was 10/25/2020 to 11/03/2022, and for Cohort 2, it was 3/24/2021 to 5/2/2021. During the intense phase, anterior nasal specimens and blood were collected during each visit. For both cohorts, the tail phase consisted of monthly visits, with respiratory specimens collected at each visit and blood specimens for serology collected every other visit. For cohort 2, additional blood specimens for peripheral blood mononuclear cells (PBMCs) were attempted at enrollment, 6 months, post booster, and at evaluation completion.
(PPTX)

S2 Fig Longitudinal antibody responses for each nursing home resident (Georgia, December 2020–July 2022; n = 37). Footnotes: Participants who were identified to have moderate or severely immunocompromising condition (n = 20) were: 1, 6, 7, 8, 10, 11, 12, 13, 17, 20, 22, 24, 25, 26, 28, 31, 32, 35, 36, 37. A moderate or severely immunocompromising condition included the following: recent or active malignancy, bone marrow transplant, solid organ transplant, primary or secondary immune deficiency, or the use of oral or intravenous steroids for more than a month or any immunosuppressant drugs. **A**: Anti-SARS-CoV-2 Spike (S) IgG. anti-S IgG: anti-SARS-CoV-2 Spike IgG; BAU/mL: Binding antibody units/mL. Y-axis: Antibody levels in BAU/mL in logarithmic scale. Footnotes: This graph shows the titers of measured anti-S IgG antibodies. Seropositivity thresholds were defined by the manufacturer and listed in the kit insert as follows: anti-S IgG 17.66 BAU/mL (lowermost dashed line). Calibration of the SARS-CoV-2 antibody assays to the 1^st^ WHO international standard for anti-SARS-CoV-2 Ig allowed us to visually assess antibody concentrations in our evaluation to those associated with a computed average overall protective threshold of 154 BAU/mL for wild type, 95% Pfizer BNT162b2 VE against COVID-19 (for two doses against wild type 530 anti-S IgG BAU/mL; Goldblatt, 2022) and 90% Moderna mRNA-1273 VE against COVID-19 (for two dose against wild type, 298 anti-S IgG BAU/mL and 775 anti-RBD IgG BAU/mL; Gilbert, 2022)—as indicated by the three upper dashed lines. **B**: Anti-SARS-CoV-2 Receptor Binding Domain (RBD) IgG. anti-RBD IgG: anti-SARS-CoV-2 Receptor Binding Domain IgG; BAU/mL: Binding antibody units/mL. Y-axis: Antibody levels in BAU/mL in logarithmic scale. Footnote: This graph shows the titers of measured anti-RBD IgG antibodies. Seropositivity thresholds were defined by the manufacturer and listed in the kit insert as follows: anti-RBD IgG 14.64 BAU/mL as indicated by the lower dashed line. Calibration of the SARS-CoV-2 antibody assays to the WHO 1^st^ international standard for anti-SARS-CoV-2 immunoglobulin allowed us to compare antibody concentrations in our evaluation to those associated with 90% Moderna mRNA-1273 VE against COVID-19 (775 anti-RBD IgG BAU/mL—as indicated by the upper dashed line; Gilbert, 2022). **C**: Anti-SARS-CoV-2 Nucleocapsid (N) IgG for those with hybrid immunity. Footnotes: anti-N IgG: anti-SARS-CoV-2 Nucleocapsid IgG; BAU/mL: Binding antibody units/mL. Y-axis: Antibody levels in BAU/mL in logarithmic scale. Footnote: This graph shows the titers of measured anti-N IgG antibodies. **D**: Percent Spike Inhibition (virus neutralization capacity). Footnote: Virus neutralizing capacity = percent spike inhibition; Y axis in %.
(PPTX)

S3 Fig **A**: Percent (%) Spike IgA Memory B Cells (MBC) in a subset of nursing home residents—Georgia, December 2020–July 2022; n = 15. The last exposure type is represented by a blue dot (SARS-CoV-2 infection) and a black dot (mRNA COVID-19 vaccine). X-axis: Time since an mRNA COVID-19 vaccine dose. Y-axis: Percent (%) Spike IgA Memory B Cells (MBC). **B**: Percent (%) Nucleocapsid IgG Memory B Cells (MBC) in a subset of nursing home residents—Georgia, December 2020–July 2022; n = 15. The last exposure type is represented by a blue dot (SARS-CoV-2 infection) and a black dot (mRNA COVID-19 vaccine). X-axis: Time since an mRNA COVID-19 vaccine dose. Y-axis: Percent (%) Nucleocapsid IgG Memory B Cells (MBC). **C**: Percent (%) Nucleocapsid IgA Memory B Cells (MBC) in a subset of nursing home residents—Georgia, December 2020–July 2022; n = 15. The last exposure type is represented by a blue dot (SARS-CoV-2 infection) and a black dot (mRNA COVID-19 vaccine). X-axis: Time since an mRNA COVID-19 vaccine dose. Y-axis: Percent (%) Nucleocapsid IgA Memory B Cells (MBC).
(PPTX)

S1 Table Definitions for spike exposures. *Spike exposure is defined as exposure to the viral spike protein due to either a SARS-CoV-2 infection(s) or a dose(s) of an mRNA COVID-19 vaccine receipt. The primary series was considered as two spike exposures. †Hybrid immunity was defined as the immune protection in individuals who have had one or more doses of an mRNA COVID-19 vaccine and have evidence of at least one SARS-CoV-2 infection before or after vaccination initiation. ‡Vaccine-induced immunity was defined as the immune protection in infection-naive individuals who have had one or more doses of an mRNA COVID-19 vaccine and remained infection-naive after vaccination initiation.
(DOCX)

S2 Table Geometric mean titer, range, and IQR for anti-spike IgG, anti-RBD IgG, anti-N IgG, and virus neutralizing capacity, by spike exposure and binned time periods—Georgia, December 2020– July 2022, n = 37. *Spike exposure is defined as exposure to the viral spike protein due to either a SARS-CoV-2 infection(s) or a dose(s) of an mRNA COVID-19 vaccine receipt. The primary series was considered as two spike exposures. †A SARS-CoV-2 infected participant was defined as a participant with infection documented in the electronic health record or confirmed by laboratory testing using real-time reverse-transcriptase polymerase chain reaction (rRT-PCR), point of care BinaxNOW^™^ COVID-19 Ag Card antigen test (BinaxNOW), or seroconversion as indicated by the presence of anti-nucleocapsid antibody (anti-N) IgG titer to SARS-CoV-2 above the cut-off for seropositivity using Meso Scale Discovery (MSD) immunoassay (MSD; Rockville, MD, USA). ‡An infection-naïve participant was defined as having an absence of a documented SARS-CoV-2 infection and negative SARS-CoV-2 laboratory test results, including seronegative for anti-N antibody. §Hybrid immunity was defined as the immune protection in individuals who have had one or more doses of an mRNA COVID-19 vaccine and have evidence of at least one SARS-CoV-2 infection before or after vaccination initiation.
(XLSX)

S3 Table Range of SARS-CoV-2% memory B cells among participants with hybrid immunity, by number of spike exposure*—Georgia, December 2020– July 2022, n = 15. *Spike exposure is defined as exposure to the viral spike protein due to either a SARS-CoV-2 infection(s) or a dose(s) of an mRNA COVID-19 vaccine receipt. The primary series was considered as two spike exposures. †Hybrid immunity was defined as the immune protection in individuals who have had one or more doses of an mRNA COVID-19 vaccine and have evidence of at least one SARS-CoV-2 infection before or after vaccination initiation. ‡ A moderate or severely immunocompromising condition included the following: recent or active malignancy, bone marrow transplant, solid organ transplant, primary or secondary immune deficiency, or the use of oral or intravenous steroids for more than a month or any immunosuppressant drugs.
(XLSX)
