# Supplementary material for: Immune response kinetics to SARS-CoV-2 infection and COVID-19 vaccination among nursing home residents—Georgia, October 2020–July 2022
Source: PLoS One. 2024 Apr 16;19(4):e0301367. doi: 10.1371/journal.pone.0301367 (PMC11020945; doi:10.1371/journal.pone.0301367)
Supplement: S1 Text — (DOCX) [file pone.0301367.s001.docx]

**Supplemental Methods:**

**Population and evaluation design:**

During each visit, electronic medical chart abstraction was performed to obtain supplemental information on past and recent medical history, medications, laboratory test results (e.g., SARS-CoV-2), clinical outcomes, and vaccine administration (including influenza). Standardized questionnaires were completed during each visit, including information about the adverse events post-COVID-19 vaccine receipt.

**Cohort 1**

In October 2020, 10 SARS-CoV-2-positive participants were enrolled from a 149-bed nursing home (Facility A) experiencing a SARS-CoV-2 outbreak. Participants were tested for SARS-CoV-2- using BinaxNOW™ COVID-19 Ag Cards (BinaxNOW) (Abbott; Scarborough, ME). The enrollment period was October 25, 2020, through November 3, 2020, and the evaluation ended in January 2022. Upon enrollment, the first four visits were conducted every other day between Oct 25, 2020, through November 9, 2020 [1] (i.e., Intense Phase); subsequent visits were monthly (i.e., Tail Phase) until January 2022 (*S1 Fig*). For the first four visits, paired bilateral anterior nasal (AN) specimens were collected for testing by BinaxNOW and real-time reverse transcription polymerase chain reaction (rRT-PCR). During monthly visits #5 through #8, one AN specimen was collected for rRT-PCR. For monthly visits #9 onwards, paired bilateral AN specimens were collected for testing by BinaxNOW and rRT-PCR. Blood specimens were collected for serology during follow-up visits at a cadence every other month for the tail phase. Vaccination clinic dates for the primary series of mRNA COVID-19 vaccine ranged from January 6, 2021, to February 24, 2021.

**Fig S1: Timeline of evaluation in nursing home residents (N=37) by cohort (n=2) and facility (n=3) —Georgia, October 2020–July 2022**

Footnotes:

Cohort 1 (n=10), Cohort 2 (n=27), and facility (n=3)

Note that for Cohort 1, the intense phase consisted of 4 visits conducted every other day for the first 10 days; for Cohort 2, the intense phase consisted of 4 visits conducted every other week for 2 months. During the intense phase, anterior nasal specimens and blood were collected during each visit. For both cohorts, the tail phase consisted of monthly visits, with respiratory specimens collected at each visit and blood specimens for serology collected every other visit. For cohort 2, additional blood specimens for peripheral blood mononuclear cells (PBMCs) were attempted at enrollment, 6 months, post booster, and at evaluation completion.

**Cohort 2**

Participant enrollment began in Facility B (150 beds) in March of 2021, Facility C (250 beds) in April of 2021, and among residents of Facility A in May of 2021. Upon completion of enrollment, visits were conducted every two weeks for the first two months of the evaluation (i.e., Intense Phase) at all three facilities. During this Intense Phase, blood specimens were collected for serology, and paired bilateral AN specimens were collected for testing by BinaxNOW and rRT-PCR. Attempts were made to collect additional blood for PBMC isolation from a subset of participants, once during the Intense Phase (dependent on the timing of enrollment and second dose of vaccination) and then at six months, post-booster, and at the end of the evaluation. During the follow-up visits (i.e., Tail Phase), blood specimens for serology were only collected every other month. In contrast, respiratory specimens for BinaxNOW testing and rRT-PCR were collected every month (*S1* *Fig*).

Vaccination dates for the first dose of mRNA COVID-19 vaccine ranged from December 20, 2020, to May 10, 2021. For the second dose, vaccination dates ranged from January 20, 2021, to June 10, 2021. Facilities A and B operated vaccine clinics for their residents, and Facility C vaccinated their residents in batches (i.e., rolling vaccination) as doses became available through their pharmacy provider. The first booster doses were provided from October 14, 2021, to February 2, 2022. Second booster doses were provided on May 20, 2022.

**Definitions**

**Supplemental Table S1: Definitions for spike exposures**

| **Spike exposures*** |  | **Hybrid Immunity†** | **Vaccine-induced immunity among the infection-naive‡** |
| --- | --- | --- | --- |
| 1 | Evidence of first infection |  | The first dose of the primary series |
| 2 |  | Evidence of first infection and the first dose of the primary series of mRNA COVID-19 vaccine | Two doses of the primary series of mRNA COVID-19 vaccine |
| 3 |  | Evidence of first infection and two doses of the primary series of mRNA COVID-19 vaccine | Primary series plus third dose (monovalent booster) of mRNA COVID-19 vaccine |
| 4 |  | Evidence of first infection and two doses of the primary series of mRNA COVID-19 vaccine and known reinfection, **OR** | Primary series and two monovalent boosters of mRNA COVID-19 vaccine. No participants in this category |
|  |  | Evidence of first infection and two doses of the primary series plus third dose (booster) of mRNA COVID-19 vaccine |  |
| 5 |  | Evidence of first infection with primary series of mRNA COVID-19 vaccine plus two boosters **OR e**vidence of first infection with primary series of mRNA COVID-19 vaccine and reinfection |  |
| 6 |  | Evidence of first infection with primary series plus two boosters of mRNA COVID-19 vaccine and evidence of reinfection |  |

Footnote: *Spike exposure is defined as exposure to the viral spike protein due to either a SARS-CoV-2 infection(s) or a dose(s) of an mRNA COVID-19 vaccine receipt. The primary series was considered as two spike exposures.

†Hybrid immunity was defined as the immune protection in individuals who have had one or more doses of an mRNA COVID-19 vaccine and have evidence of at least one SARS-CoV-2 infection before or after vaccination initiation.

‡Vaccine-induced immunity was defined as the immune protection in infection-naive individuals who have had one or more doses of an mRNA COVID-19 vaccine and remained infection-naive after vaccination initiation.

A moderate or severely immunocompromising condition included the following: recent or active malignancy, bone marrow transplant, solid organ transplant, primary or secondary immune deficiency, or the use of oral or intravenous steroids for more than a month or any immunosuppressant drugs.

**Statistical analysis:**

Evaluation data were collected and managed using REDCap (Research Electronic Data Capture) electronic data capture tools hosted at CDC [2, 3]. It provides an interface for validated data capture, audit trails for tracking data manipulation and export procedures, automated export procedures for data downloads to common statistical packages, and procedures for data integration and interoperability with external sources.

We describe participants’ demographic and clinical characteristics by frequencies and proportions for categorical data and by means and ranges for continuous data. Univariate analyses were performed on infection status, type of mRNA COVID-19 vaccine, age, sex, race, and comorbidities. Analysis was not performed on ethnicity due to low power, as only one participant self-reported as Hispanic.

Due to the reduction in sample size in the lower exposure groups, we only performed modeling on the following: 2 exposures among hybrid and vaccine-induced immunity groups, 3 exposures among hybrid and vaccine-induced immunity groups, 3 exposures among hybrid immunity compared to 4 exposures among hybrid immunity, and 4 exposures among hybrid immunity compared to 5 exposures among hybrid immunity (*S1* *Table*).

Due to a limited number of available peripheral blood mononuclear cells (PBMCs) samples, no statistical analysis was conducted, and the percent of anti-S-specific MBC totals are shown.

**Laboratory:**

CDC staff tested paired bilateral AN specimens using BinaxNOW point-of-care antigen tests at the bedside and rRT-PCR [4]. All ANs were collected per CDC guidelines for specimen collection and handling [5]. Details of specimen collection, BinaxNOW, and rRT-PCR were described previously [1],[6]. The respiratory specimens also underwent RT-PCR testing for influenza.

Although whole genome sequencing was not performed on specimens collected during this evaluation period, as per the information shared by the Georgia Department of Public Health (email correspondence), it is possible that this period covered the wild type [March 2020], alpha (B.1.1.7) [December 2020], Epsilon (B.1.427) [December 2020], Eta (B.1.525) [January 2021], Zeta (P.2) [January 2021], beta (B.1.351) [February 2021], Kappa (B.1.617.1) [March 2021], Gamma (P.1) [March 2021], Mu (B.1.621) [April 2021], Delta (B.1.617.2) [April 2021], Iota (B.1.526) [April 2021] and Omicron (B.1.1.529) [November 2021] waves based on the detection in Georgia.

**Serology testing**

Overall, 322 plasma specimens for serology from 37 participants were collected and resulted during this evaluation period: a range of 4–12 specimens per participant. Specimens for serology were collected a mean of 32 days (range 0–89 days) after the completion of the mRNA COVID-19 vaccine primary series but before the first monovalent booster dose, a mean of 20 days (range 8–78 days) after the first monovalent booster but before second monovalent booster, and 13 days after the second monovalent booster.

**Memory B cells**

Whole blood tubes were centrifuged at 400 x g for 30 min at room temperature with no break. Mononuclear cells were collected, washed with Roswell Park Memorial Institute (RPMI) 1640 medium, counted, then frozen in 10% dimethyl sulfoxide (DMSO) in fetal bovine serum at -150 ± 15 °C. After thawing, PBMCs were stimulated with a cocktail of Pokeweed mitogen (1/1000 of 1 mg/ml, sigma), Staphylococcus aureus Cowan (1 1/10,000 Sigma), and CpG-2006 oligonucleotide (6 µg/ml Invivogen). The cultures were then incubated for 6 days at 37°C in 5% CO_2_, harvested, washed, and added to plates coated with affinity-purified goat anti-human IgA+IgG+IgM (H+L) (Jackson Immunoresearch) or SARS-CoV-2 spike protein (stabilized ectodomain expressed in HEK-293 cells and purified in-lab). Both total and antigen-specific IgG- or IgA-secreting memory B cells were detected using biotinylated donkey anti-human IgG (Jackson Immunoesearch, West Grove, PA) or biotinylated goat anti-human IgA (Southern Biotech), respectively. Spots were enumerated with an Immunospot® reader (Cellular Technology, Cleveland, OH). The frequency of antigen-specific B cells was reported as the percent of total IgG memory B cells per million PBMCs. The S protein used in this assay was highly specific for SARS-CoV-2-specific MBC detection based on pre-pandemic negative controls (data not shown) [7]. Overall, 30 whole blood specimens for PBMC testing from 15 participants were collected and resulted during this evaluation period, a range of 1-4 specimens per participant.

**Supplemental Results:**

We did not detect statistically significant differences in the percentage decline of anti-S IgG or neutralizing antibodies by age, sex, vaccine type, immunocompromised status, or comorbid conditions when performing linear mixed effects modeling among those with 3 spike exposures in the hybrid immunity group (Table 2).

Of the 37 participants, 20 (54%) were identified as having moderately or severely immunocompromising conditions. These participants did not receive an additional vaccine dose after the primary series, as recommended for immunocompromised individuals. Among those with 3 exposures (n=17), the peak anti-S IgG titer was 6710.4 BAU/mL with a decline of 24.4% decline per month, while in the non-immunocompromised (n=14), the peak anti-S IgG titer was 4410.796 BAU/mL with 21.0% decline per month; there was no statistical difference in their decline (p=0.2). In a subset of participants with PBMC collections, we observed that participants with hybrid immunity who had a moderate or severely immunocompromising condition were able to elicit an MBC response; an increase in %S IgG was observed with each spike exposure, similar to the non-immunocompromised participants with hybrid immunity, albeit lower %S IgG response (*S3 Table*). Participants with vaccine-only induced immunity were able to elicit an MBC %S IgG response, but we were unable to compare due to the very small numbers.

Although the paired bilateral AN specimens were collected, as the focus of this evaluation was on antibody response, the timing of collection may have affected the outcome of the rRT-PCR testing, such that it may have resulted in insufficient virus for sequencing. Thus, we relied on the BinaxNOW point-of-care antigen tests at the bedside and the facilities’ test results. Influenza RT-PCR testing was performed throughout this evaluation period. None of the participants developed or were diagnosed with influenza during this evaluation period.

**Supplemental Figs and tables**

**Fig S1: Timeline of evaluation in nursing home residents (N=37) by cohort (n=2) and facility (n=3) —Georgia, October 2020–July 2022**

Footnotes:

Cohort 1 (n=10), Cohort 2 (n=27), and facility (n=3)

Note that for Cohort 1, the intense phase consisted of 4 visits conducted every other day for the first 10 days; for Cohort 2, the intense phase consisted of 4 visits conducted every other week for 2 months. The enrollment period for Cohort 1 was 10/25/2020 to 11/03/2022 and for Cohort 2, it was 3/24/2021 to 5/2/2021. During the intense phase, anterior nasal specimens and blood were collected during each visit. For both cohorts, the tail phase consisted of monthly visits, with respiratory specimens collected at each visit and blood specimens for serology collected every other visit. For cohort 2, additional blood specimens for peripheral blood mononuclear cells (PBMCs) were attempted at enrollment, 6 months, post booster, and at evaluation completion.

**Fig S2: Longitudinal antibody responses for each nursing home resident (Georgia, December 2020–July 2022; n=37)**

Footnotes:

Participants who were identified to have moderate or severely immunocompromising condition (n=20) were: 1, 6, 7, 8, 10, 11, 12, 13, 17, 20, 22, 24, 25, 26, 28, 31, 32, 35, 36, 37.

A moderate or severely immunocompromising condition included the following: recent or active malignancy, bone marrow transplant, solid organ transplant, primary or secondary immune deficiency, or the use of oral or intravenous steroids for more than a month or any immunosuppressant drugs.

**Fig S2A: Anti-SARS-CoV-2 Spike (S) IgG**

anti-S IgG: anti-SARS-CoV-2 Spike IgG; BAU/mL: Binding antibody units/mL

Y-axis: Antibody levels in BAU/mL in logarithmic scale

This graph shows the titers of measured anti-S IgG antibodies.

Seropositivity thresholds were defined by the manufacturer and listed in the kit insert as follows: anti-S IgG 17.66 BAU/mL (lowermost dashed line).

Calibration of the SARS-CoV-2 antibody assays to the 1^st^ WHO international standard for anti-SARS-CoV-2 Ig allowed us to visually assess antibody concentrations in our evaluation to those associated with a computed average overall protective threshold of 154 BAU/mL for wild type, 95% Pfizer BNT162b2 VE against COVID-19 (for two doses against wild type 530 anti-S IgG BAU/mL; Goldblatt, 2022) and 90% Moderna mRNA-1273 VE against COVID-19 (for two dose against wild type, 298 anti-S IgG BAU/mL and 775 anti-RBD IgG BAU/mL; Gilbert, 2022) — as indicated by the three upper dashed lines.

**Fig S2B: Anti-SARS-CoV-2 Receptor Binding Domain (RBD) IgG**

anti-RBD IgG: anti-SARS-CoV-2 Receptor Binding Domain IgG; BAU/mL: Binding antibody units/mL

Y-axis: Antibody levels in BAU/mL in logarithmic scale

This graph shows the titers of measured anti-RBD IgG antibodies.

Seropositivity thresholds were defined by the manufacturer and listed in the kit insert as follows: anti-RBD IgG 14.64 BAU/mL as indicated by the lower dashed line. Calibration of the SARS-CoV-2 antibody assays to the WHO 1^st^ international standard for anti-SARS-CoV-2 immunoglobulin allowed us to compare antibody concentrations in our evaluation to those associated with 90% Moderna mRNA-1273 VE against COVID-19 (775 anti-RBD IgG BAU/mL — as indicated by the upper dashed line; Gilbert, 2022).

**Fig S2C: Anti-SARS-CoV-2 Nucleocapsid (N) IgG for those with hybrid immunity**

Footnotes:

anti-N IgG: anti-SARS-CoV-2 Nucleocapsid IgG; BAU/mL: Binding antibody units/mL

Y-axis: Antibody levels in BAU/mL in logarithmic scale

This graph shows the titers of measured anti-N IgG antibodies.

**Fig S2D: Percent Spike Inhibition (virus neutralization capacity)**

Footnote:

Virus neutralizing capacity = percent spike inhibition; Y axis in %

**Fig S3A: Percent (%) Spike IgA Memory B Cells (MBC) in a subset of nursing home residents —Georgia, December 2020–July 2022; n=15**

The last exposure type is represented by a blue dot (SARS-CoV-2 infection) and a black dot (mRNA COVID-19 vaccine)

X-axis: Time since an mRNA COVID-19 vaccine dose

Y-axis: Percent (%) Spike IgA Memory B Cells (MBC)

**Fig S3B: Percent (%) Nucleocapsid IgG Memory B Cells (MBC) in a subset of nursing home residents —Georgia, December 2020–July 2022; n=15**

The last exposure type is represented by a blue dot (SARS-CoV-2 infection) and a black dot (mRNA COVID-19 vaccine)

X-axis: Time since an mRNA COVID-19 vaccine dose

Y-axis: Percent (%) Nucleocapsid IgG Memory B Cells (MBC)

**Fig S3C: Percent (%) Nucleocapsid IgA Memory B Cells (MBC) in a subset of nursing home residents—Georgia, December 2020–July 2022; n=15**

The last exposure type is represented by a blue dot (SARS-CoV-2 infection) and a black dot (mRNA COVID-19 vaccine)

X-axis: Time since an mRNA COVID-19 vaccine dose

Y-axis: Percent (%) Nucleocapsid IgA Memory B Cells (MBC)

**Table S2:** Supplemental Table 2. Geometric Mean Titer, Range, and IQR for Anti-Spike IgG, Anti-RBD IgG, Anti-N IgG, and Virus Neutralizing Capacity, by Spike Exposure and Binned Time Periods – Georgia, December 2020– July 2022, n=37

Footnote:

*Spike exposure is defined as exposure to the viral spike protein due to either a SARS-CoV-2 infection(s) or a dose(s) of an mRNA COVID-19 vaccine receipt. The primary series was considered as two spike exposures.

†A SARS-CoV-2 infected participant was defined as a participant with infection documented in the electronic health record or confirmed by laboratory testing using real-time reverse-transcriptase polymerase chain reaction (rRT-PCR), point of care BinaxNOW^TM^ COVID-19 Ag Card antigen test (BinaxNOW), or seroconversion as indicated by the presence of anti-nucleocapsid antibody (anti-N) IgG titer to SARS-CoV-2 above the cut-off for seropositivity using Meso Scale Discovery (MSD) immunoassay (MSD; Rockville, MD, USA).

‡An infection-naïve participant was defined as having an absence of a documented SARS-CoV-2 infection and negative SARS-CoV-2 laboratory test results, including seronegative for anti-N antibody.

§Hybrid immunity was defined as the immune protection in individuals who have had one or more doses of an mRNA COVID-19 vaccine and have evidence of at least one SARS-CoV-2 infection before or after vaccination initiation

**Table S3**: Range of SARS-CoV-2 % Memory B Cells Among Participants with Hybrid Immunity, By Number of Spike Exposure* – Georgia, December 2020– July 2022, n=15

Footnote:

*Spike exposure is defined as exposure to the viral spike protein due to either a SARS-CoV-2 infection(s) or a dose(s) of an mRNA COVID-19 vaccine receipt. The primary series was considered as two spike exposures.

†Hybrid immunity was defined as the immune protection in individuals who have had one or more doses of an mRNA COVID-19 vaccine and have evidence of at least one SARS-CoV-2 infection before or after vaccination initiation.

‡ A moderate or severely immunocompromising condition included the following: recent or active malignancy, bone marrow transplant, solid organ transplant, primary or secondary immune deficiency, or the use of oral or intravenous steroids for more than a month or any immunosuppressant drugs.

**Supplemental References:**

1. Moritz ED, McKay SL, Tobolowsky FA, LaVoie SP, Waltenburg MA, Lecy KD, et al. Repeated antigen testing among severe acute respiratory coronavirus virus 2 (SARS-CoV-2)–positive nursing home residents. Infection Control & Hospital Epidemiology. 2021:1-4.

2. Harris PA, Taylor R, Thielke R, Payne J, Gonzalez N, Conde JG. Research electronic data capture (REDCap)—A metadata-driven methodology and workflow process for providing translational research informatics support. Journal of Biomedical Informatics. 2009;42(2):377-81.

3. Harris PA, Taylor R, Minor BL, Elliott V, Fernandez M, O'Neal L, et al. The REDCap consortium: Building an international community of software platform partners. Journal of Biomedical Informatics. 2019;95:103208.

4. CDC. SARS-CoV-2 antigen testing in long term care facilities. 2022 [Available from: <https://www.cdc.gov/coronavirus/2019-ncov/lab/resources/antigen-tests-guidelines.html.11/02/2023>

5. CDC. Interim Guidelines for Collecting and Handling of Clinical Specimens for COVID-19 Testing: CDC; 2022 [updated 07/15/2022. Available from: <https://www.cdc.gov/coronavirus/2019-ncov/lab/guidelines-clinical-specimens.html#:~:text=Store%20respiratory%20specimens%20at%202,70%C2%B0C%20or%20lower.11/02/2023>

6. McKay SL, Tobolowsky FA, Moritz ED, Hatfield KM, Bhatnagar A, LaVoie SP, et al. Performance Evaluation of Serial SARS-CoV-2 Rapid Antigen Testing During a Nursing Home Outbreak. Ann Intern Med. 2021;174(7):945-51.

7. Shah MM, Rasheed MAU, Harcourt JL, Abedi GR, Stumpf MM, Kirking HL, et al. Twelve-Month Follow-up of Early COVID-19 Cases in the United States: Cellular and Humoral Immune Longevity. Open Forum Infect Dis. 2022;9(3):ofab664.
