# Supplementary material for: Immune response kinetics to SARS-CoV-2 infection and COVID-19 vaccination among nursing home residents—Georgia, October 2020–July 2022
Source: PLoS One. 2024 Apr 16;19(4):e0301367. doi: 10.1371/journal.pone.0301367 (PMC11020945; doi:10.1371/journal.pone.0301367)
Supplement: S1 Fig — Cohort 1 (n = 10), Cohort 2 (n = 27), and facility (n = 3). Note that for Cohort 1, the intense phase consisted of 4 visits conducted every other day for the first 10 days; for Cohort 2, the intense phase consisted of 4 visits conducted every other week for 2 months. The enrollment period for Cohort 1 was 10/25/2020 to 11/03/2022, and for Cohort 2, it was 3/24/2021 to 5/2/2021. During the intense phase, anterior nasal specimens and blood were collected during each visit. For both cohorts, the tail phase consisted of monthly visits, with respiratory specimens collected at each visit and blood specimens for serology collected every other visit. For cohort 2, additional blood specimens for peripheral blood mononuclear cells (PBMCs) were attempted at enrollment, 6 months, post booster, and at evaluation completion. (PPTX) [file pone.0301367.s003.pptx]

## Slide 1
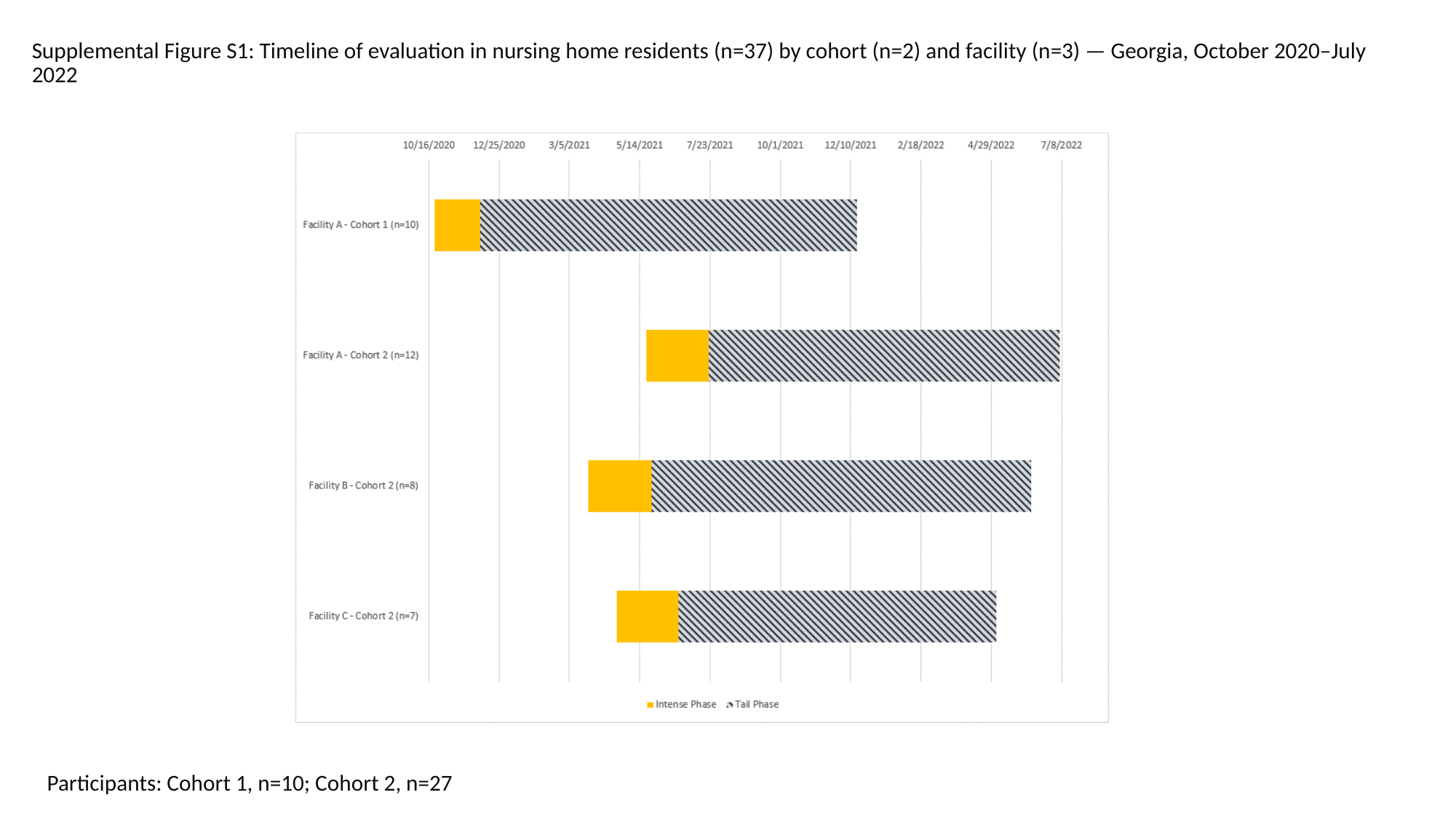

# Supplemental Figure S1: Timeline of evaluation in nursing home residents (n=37) by cohort (n=2) and facility (n=3) — Georgia, October 2020–July 2022
Participants: Cohort 1, n=10; Cohort 2, n=27
