# Supplementary material for: Immune response kinetics to SARS-CoV-2 infection and COVID-19 vaccination among nursing home residents—Georgia, October 2020–July 2022
Source: PLoS One. 2024 Apr 16;19(4):e0301367. doi: 10.1371/journal.pone.0301367 (PMC11020945; doi:10.1371/journal.pone.0301367)
Supplement: S2 Fig — Footnotes: Participants who were identified to have moderate or severely immunocompromising condition (n = 20) were: 1, 6, 7, 8, 10, 11, 12, 13, 17, 20, 22, 24, 25, 26, 28, 31, 32, 35, 36, 37. A moderate or severely immunocompromising condition included the following: recent or active malignancy, bone marrow transplant, solid organ transplant, primary or secondary immune deficiency, or the use of oral or intravenous steroids for more than a month or any immunosuppressant drugs. A: Anti-SARS-CoV-2 Spike (S) IgG. anti-S IgG: anti-SARS-CoV-2 Spike IgG; BAU/mL: Binding antibody units/mL. Y-axis: Antibody levels in BAU/mL in logarithmic scale. Footnotes: This graph shows the titers of measured anti-S IgG antibodies. Seropositivity thresholds were defined by the manufacturer and listed in the kit insert as follows: anti-S IgG 17.66 BAU/mL (lowermost dashed line). Calibration of the SARS-CoV-2 antibody assays to the 1st WHO international standard for anti-SARS-CoV-2 Ig allowed us to visually assess antibody concentrations in our evaluation to those associated with a computed average overall protective threshold of 154 BAU/mL for wild type, 95% Pfizer BNT162b2 VE against COVID-19 (for two doses against wild type 530 anti-S IgG BAU/mL; Goldblatt, 2022) and 90% Moderna mRNA-1273 VE against COVID-19 (for two dose against wild type, 298 anti-S IgG BAU/mL and 775 anti-RBD IgG BAU/mL; Gilbert, 2022)—as indicated by the three upper dashed lines. B: Anti-SARS-CoV-2 Receptor Binding Domain (RBD) IgG. anti-RBD IgG: anti-SARS-CoV-2 Receptor Binding Domain IgG; BAU/mL: Binding antibody units/mL. Y-axis: Antibody levels in BAU/mL in logarithmic scale. Footnote: This graph shows the titers of measured anti-RBD IgG antibodies. Seropositivity thresholds were defined by the manufacturer and listed in the kit insert as follows: anti-RBD IgG 14.64 BAU/mL as indicated by the lower dashed line. Calibration of the SARS-CoV-2 antibody assays to the WHO 1st international standard for anti-SARS-CoV [file pone.0301367.s004.pptx]

## Slide 1
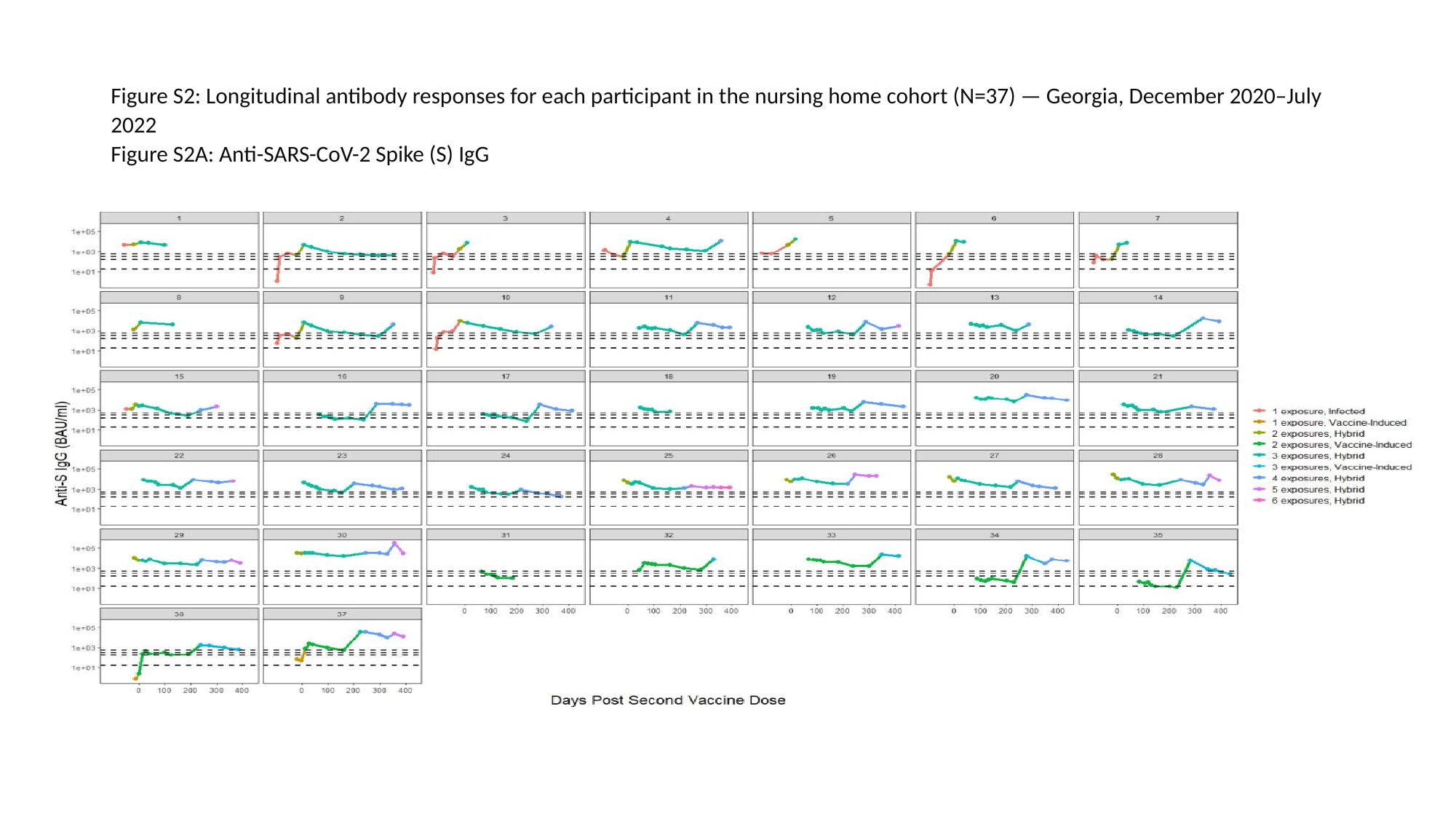

# Figure S2: Longitudinal antibody responses for each participant in the nursing home cohort (N=37) — Georgia, December 2020–July 2022 Figure S2A: Anti-SARS-CoV-2 Spike (S) IgG

## Slide 2
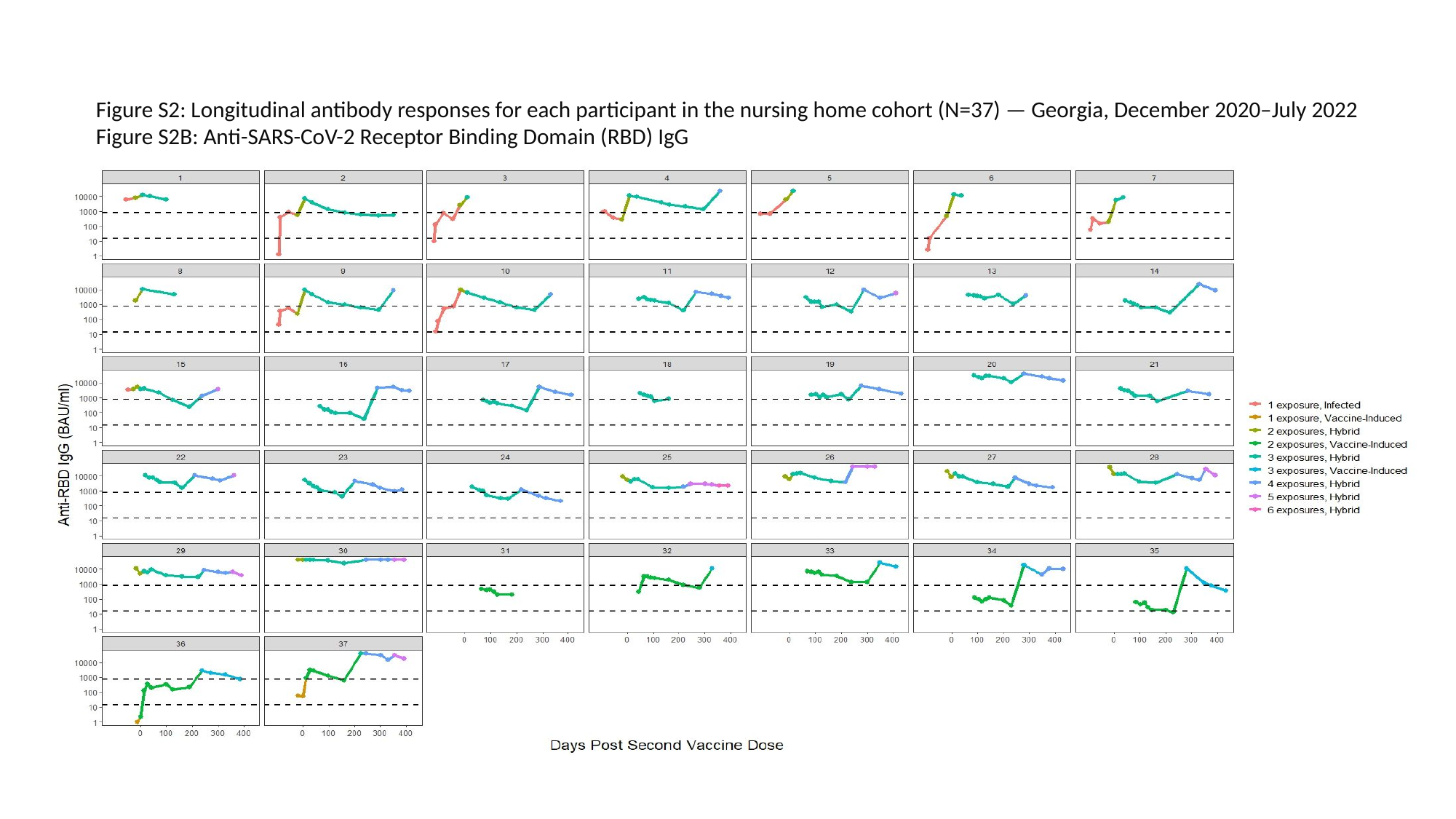

Figure S2: Longitudinal antibody responses for each participant in the nursing home cohort (N=37) — Georgia, December 2020–July 2022
Figure S2B: Anti-SARS-CoV-2 Receptor Binding Domain (RBD) IgG

## Slide 3
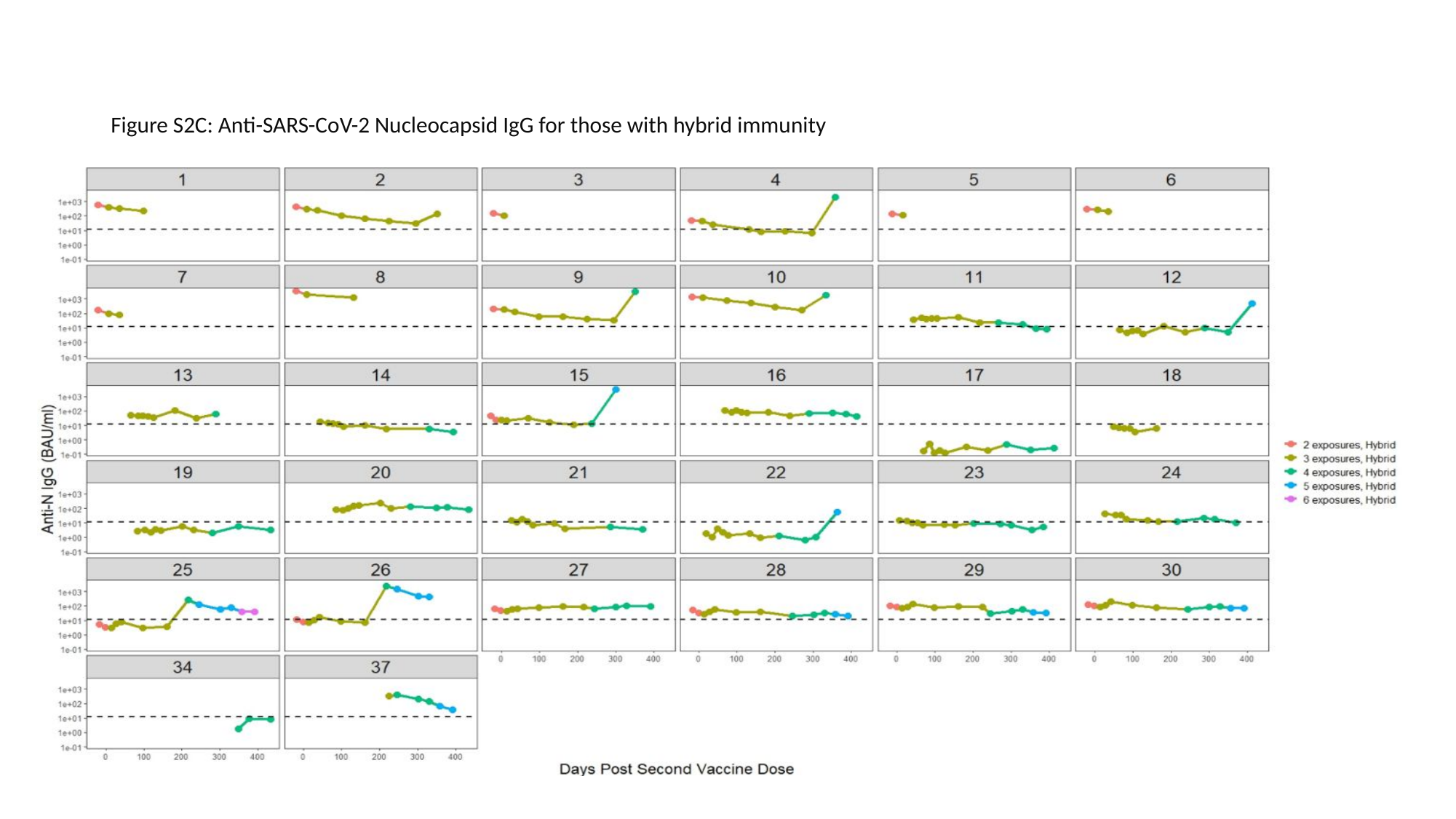

# Figure S2C: Anti-SARS-CoV-2 Nucleocapsid IgG for those with hybrid immunity

## Slide 4
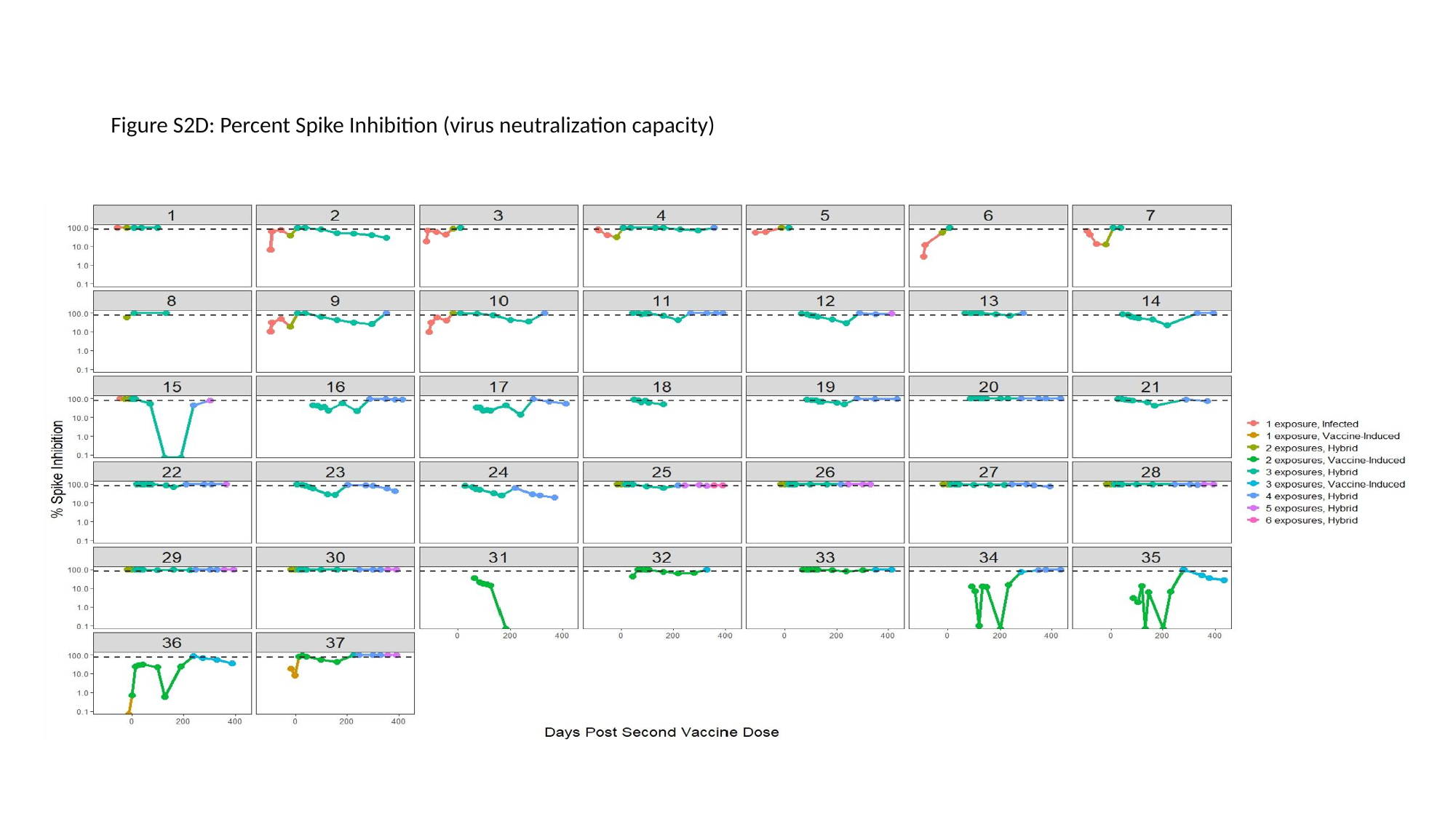

# Figure S2D: Percent Spike Inhibition (virus neutralization capacity)
