# Supplementary material for: Immune response kinetics to SARS-CoV-2 infection and COVID-19 vaccination among nursing home residents—Georgia, October 2020–July 2022
Source: PLoS One. 2024 Apr 16;19(4):e0301367. doi: 10.1371/journal.pone.0301367 (PMC11020945; doi:10.1371/journal.pone.0301367)
Supplement: S3 Fig — A: Percent (%) Spike IgA Memory B Cells (MBC) in a subset of nursing home residents—Georgia, December 2020–July 2022; n = 15. The last exposure type is represented by a blue dot (SARS-CoV-2 infection) and a black dot (mRNA COVID-19 vaccine). X-axis: Time since an mRNA COVID-19 vaccine dose. Y-axis: Percent (%) Spike IgA Memory B Cells (MBC). B: Percent (%) Nucleocapsid IgG Memory B Cells (MBC) in a subset of nursing home residents—Georgia, December 2020–July 2022; n = 15. The last exposure type is represented by a blue dot (SARS-CoV-2 infection) and a black dot (mRNA COVID-19 vaccine). X-axis: Time since an mRNA COVID-19 vaccine dose. Y-axis: Percent (%) Nucleocapsid IgG Memory B Cells (MBC). C: Percent (%) Nucleocapsid IgA Memory B Cells (MBC) in a subset of nursing home residents—Georgia, December 2020–July 2022; n = 15. The last exposure type is represented by a blue dot (SARS-CoV-2 infection) and a black dot (mRNA COVID-19 vaccine). X-axis: Time since an mRNA COVID-19 vaccine dose. Y-axis: Percent (%) Nucleocapsid IgA Memory B Cells (MBC). (PPTX) [file pone.0301367.s005.pptx]

## Slide 1
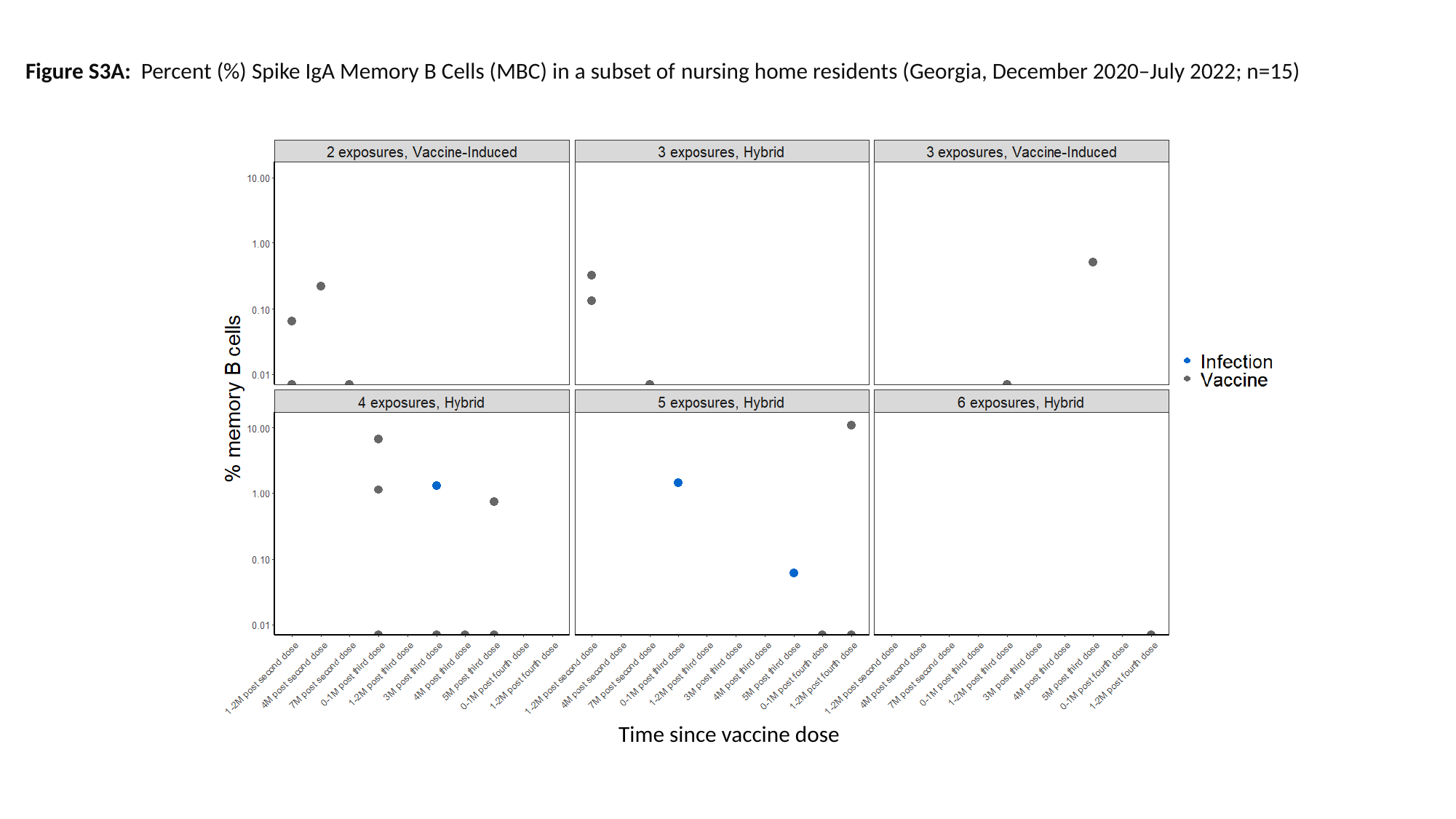

Figure S3A: Percent (%) Spike IgA Memory B Cells (MBC) in a subset of nursing home residents (Georgia, December 2020–July 2022; n=15)
Time since vaccine dose

## Slide 2
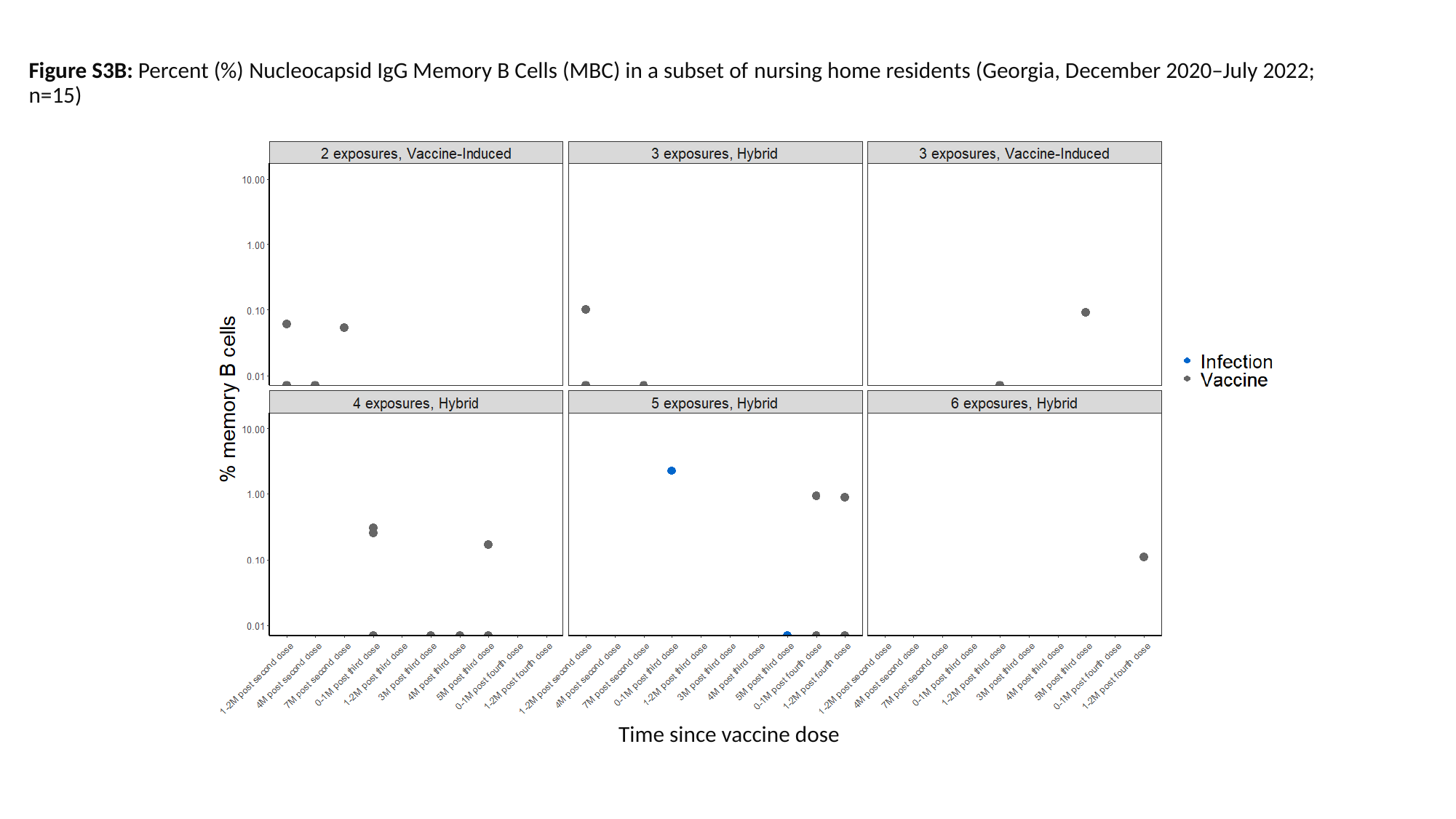

Figure S3B: Percent (%) Nucleocapsid IgG Memory B Cells (MBC) in a subset of nursing home residents (Georgia, December 2020–July 2022; n=15)
Time since vaccine dose

## Slide 3
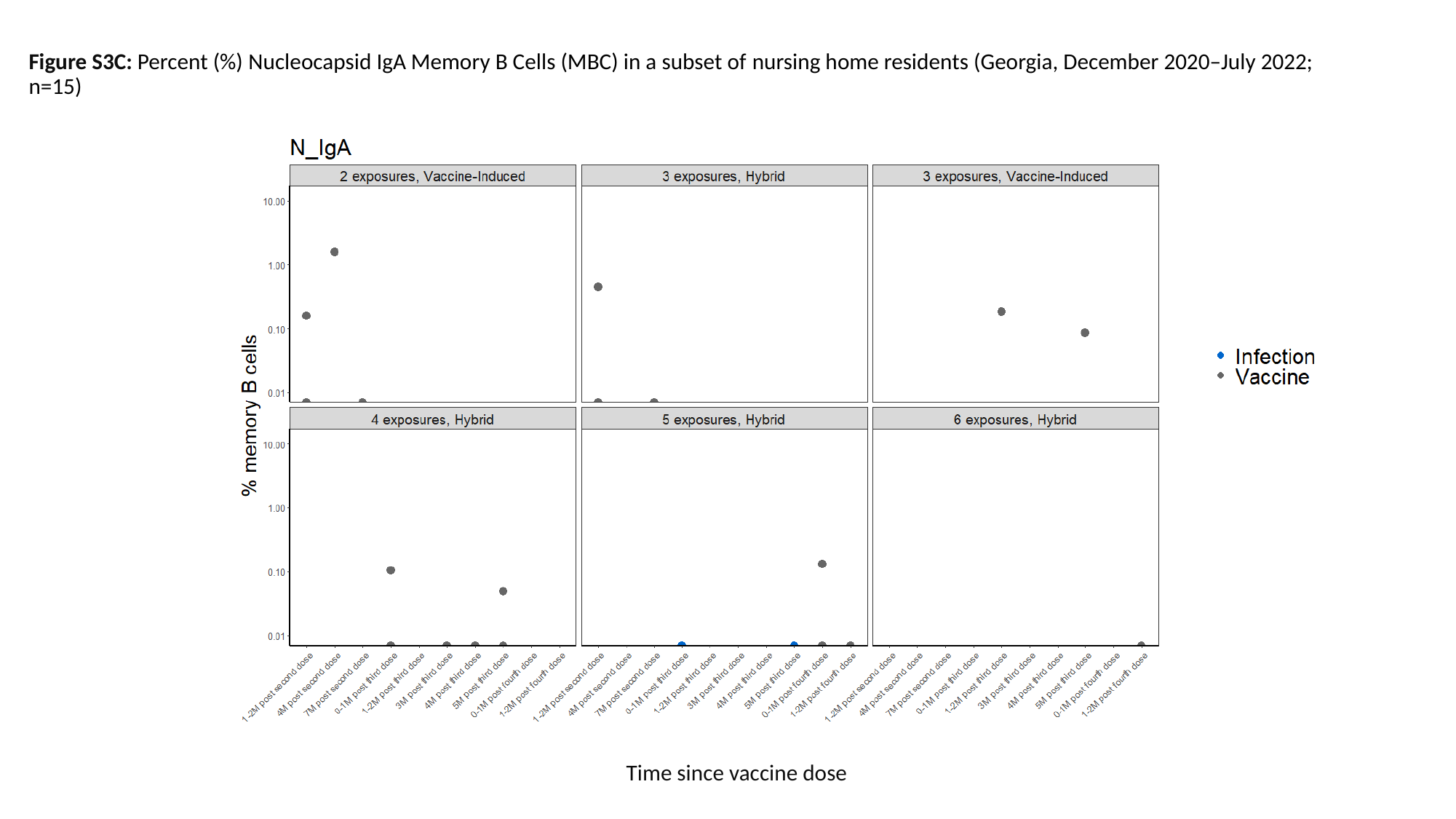

Figure S3C: Percent (%) Nucleocapsid IgA Memory B Cells (MBC) in a subset of nursing home residents (Georgia, December 2020–July 2022; n=15)
Time since vaccine dose
