# Supplementary material for: Immune response kinetics to SARS-CoV-2 infection and COVID-19 vaccination among nursing home residents—Georgia, October 2020–July 2022
Source: PLoS One. 2024 Apr 16;19(4):e0301367. doi: 10.1371/journal.pone.0301367 (PMC11020945; doi:10.1371/journal.pone.0301367)
Supplement: S1 Table — *Spike exposure is defined as exposure to the viral spike protein due to either a SARS-CoV-2 infection(s) or a dose(s) of an mRNA COVID-19 vaccine receipt. The primary series was considered as two spike exposures. †Hybrid immunity was defined as the immune protection in individuals who have had one or more doses of an mRNA COVID-19 vaccine and have evidence of at least one SARS-CoV-2 infection before or after vaccination initiation. ‡Vaccine-induced immunity was defined as the immune protection in infection-naive individuals who have had one or more doses of an mRNA COVID-19 vaccine and remained infection-naive after vaccination initiation. (DOCX) [file pone.0301367.s006.docx]

**Table S1: Definitions for spike exposures**

| **Spike exposures*** |  | **Hybrid Immunity†** | **Vaccine-induced immunity among the infection-naive‡** |
| --- | --- | --- | --- |
| 1 | Evidence of first infection |  | The first dose of the primary series |
| 2 |  | Evidence of first infection and the first dose of the primary series of mRNA COVID-19 vaccine | Two doses of the primary series of mRNA COVID-19 vaccine |
| 3 |  | Evidence of first infection and two doses of the primary series of mRNA COVID-19 vaccine | Primary series plus third dose (monovalent booster) of mRNA COVID-19 vaccine |
| 4 |  | Evidence of first infection and two doses of the primary series of mRNA COVID-19 vaccine and known reinfection, **OR** | Primary series and two monovalent boosters of mRNA COVID-19 vaccine. No participants in this category |
|  |  | Evidence of first infection and two doses of the primary series plus third dose (booster) of mRNA COVID-19 vaccine |  |
| 5 |  | Evidence of first infection with primary series of mRNA COVID-19 vaccine plus two boosters **OR e**vidence of first infection with primary series of mRNA COVID-19 vaccine and reinfection |  |
| 6 |  | Evidence of first infection with primary series plus two boosters of mRNA COVID-19 vaccine and evidence of reinfection |  |

Footnote: *Spike exposure is defined as exposure to the viral spike protein due to either a SARS-CoV-2 infection(s) or a dose(s) of an mRNA COVID-19 vaccine receipt. The primary series was considered as two spike exposures.

†Hybrid immunity was defined as the immune protection in individuals who have had one or more doses of an mRNA COVID-19 vaccine and have evidence of at least one SARS-CoV-2 infection before or after vaccination initiation.

‡Vaccine-induced immunity was defined as the immune protection in infection-naive individuals who have had one or more doses of an mRNA COVID-19 vaccine and remained infection-naive after vaccination initiation.
